# Transcriptional and Morpho-Physiological Responses of *Marchantia polymorpha* upon Phosphate Starvation

**DOI:** 10.3390/ijms21218354

**Published:** 2020-11-07

**Authors:** Félix Rico-Reséndiz, Sergio Alan Cervantes-Pérez, Annie Espinal-Centeno, Melissa Dipp-Álvarez, Araceli Oropeza-Aburto, Enrique Hurtado-Bautista, Andrés Cruz-Hernández, John L. Bowman, Kimitsune Ishizaki, Mario A. Arteaga-Vázquez, Luis Herrera-Estrella, Alfredo Cruz-Ramírez

**Affiliations:** 1 Molecular and Developmental Complexity Group, Unidad de Genómica Avanzada, Centro de Investigación y Estudios Avanzados, Instituto Politécnico Nacional, Irapuato 36824, Guanajuato, Mexico; edgardo.rico@cinvestav.mx (F.R.-R.); annie.espinal@cinvestav.mx (A.E.-C.); melissa.dipp@cinvestav.mx (M.D.-Á.); 2 Plant Physiology and Metabolic Engineering Group, Unidad de Genómica Avanzada, Centro de Investigación y Estudios Avanzados, Instituto Politécnico Nacional, Irapuato 36824, Guanajuato, Mexico; sergio.cervantes@cinvestav.mx (S.A.C.-P.); araceli.oropeza@cinvestav.mx (A.O.-A.); lherrerae@cinvestav.mx (L.H.-E.); 3 Molecular Biology and Microbial Ecology, Unidad Irapuato, Centro de Investigación y Estudios Avanzados, Instituto Politécnico Nacional, Irapuato 36824, Guanajuato, Mexico; enrique.hurtado@cinvestav.mx; 4 Escuela de Agronomía, Universidad de La Salle Bajío, León 37160, Guanajuato, Mexico; acruz@delasalle.edu.mx; 5 School of Biological Sciences, Monash University, Melbourne, Victoria 3800, Australia; john.bowman@monash.edu; 6 Graduate School of Science, Kobe University, Kobe 657-8501, Japan; kimi@emerald.kobe-u.ac.jp; 7 Group of Epigenetics and Developmental Biology, Instituto de Biotecnología y Ecología Aplicada (INBIOTECA), Universidad Veracruzana, Xalapa 91640, Mexico; maarteaga@uv.mx; 8 Institute of Genomics for Crop Abiotic Stress Tolerance, Department of Plant and Soil Sciences, Texas Tech University, Lubbock, TX 79409, USA

**Keywords:** land plant evolution, *Marchantia polymorpha*, Pi starvation and RNA-seq

## Abstract

Phosphate (Pi) is a pivotal nutrient that constraints plant development and productivity in natural ecosystems. Land colonization by plants, more than 470 million years ago, evolved adaptive mechanisms to conquer Pi-scarce environments. However, little is known about the molecular basis underlying such adaptations at early branches of plant phylogeny. To shed light on how early divergent plants respond to Pi limitation, we analyzed the morpho-physiological and transcriptional dynamics of *Marchantia polymorpha* upon Pi starvation. Our phylogenomic analysis highlights some gene networks present since the Chlorophytes and others established in the Streptophytes (e.g., PHR1–SPX1 and STOP1–ALMT1, respectively). At the morpho-physiological level, the response is characterized by the induction of phosphatase activity, media acidification, accumulation of auronidins, reduction of internal Pi concentration, and developmental modifications of rhizoids. The transcriptional response involves the induction of Mp*PHR1*, Pi transporters, lipid turnover enzymes, and Mp*MYB14*, which is an essential transcription factor for auronidins biosynthesis. Mp*STOP2* up-regulation correlates with expression changes in genes related to organic acid biosynthesis and transport, suggesting a preference for citrate exudation. An analysis of MpPHR1 binding sequences (P1BS) shows an enrichment of this *cis* regulatory element in differentially expressed genes. Our study unravels the strategies, at diverse levels of organization, exerted by *M. polymorpha* to cope with low Pi availability.

## 1. Introduction

The colonization of land by plants was a complex ecological, evolutionary, and developmental (eco-evo-devo) process that occurred around 470 million years ago [1,2]. Several developmental innovations, which allowed early plants to adapt and survive on terrestrial environments, have been deeply reviewed elsewhere [3,4,5]. However, little is known about the molecular innovations that allowed plants to thrive in conditions of low nutrient availability. The molecular mechanisms underlying the morphological and physiological strategies to forage low available nutrients have been mostly described in diverse plant root systems. This is the case for most studies focused on describing the adaptation of plants to phosphate starvation [6,7,8]. Phosphorus is an essential macronutrient for proper plant development, which can only be acquired as ortho-phosphate (Pi) by plant roots [9]. In natural ecosystems, Pi is generally distributed at low concentrations due to the rapid formation of inorganic complexes with Ca^2+^ or Al^3+^ and Fe^3+^, under alkaline or acid conditions, respectively [10,11]. This phenomenon causes up to 70% of global agricultural areas to display a limited Pi availability, affecting both plant development and crop productivity [12]. To enhance Pi uptake and recycling, diverse molecular mechanisms underlying plant morphological and physiological strategies are induced [7,8]. In *Arabidopsis thaliana*, these strategies have been characterized and classified in local and systemic responses [13]. The local response largely depends on the contact of the primary root tip with a Pi-depleted substratum, whose final outcome is the full differentiation of the primary root meristem and the triggering of lateral root development [14]. One of the early events that takes place in the local response of Arabidopsis to this stress involves the accumulation of the protein SENSITIVE TO PROTON RHIZOTOXICITY 1 (AtSTOP1), a zinc finger transcription factor (TF) [15]. AtSTOP1 promotes the expression of several genes including the *ALUMINIUM ACTIVATED MALATE TRANSPORTER 1* (*AtALMT1*), which encodes a malic acid efflux transporter [15,16]. Malate efflux contributes to the modification of the iron distribution and impacts directly the Arabidopsis root system architecture (RSA) under low Pi availability, Al^3+^ toxicity, and acid pH [15,16]. AtSTOP1 induces the expression of *REGULATION OF ALMT1 EXPRESSION 1* (*AtRAE1*), which encodes an F-Box protein that ubiquitinates STOP1, which is degraded via the 26S proteasome pathway [17]. Additionally, exuded organic acids (OAs) release Pi from complexes with Al^3+^ or Fe^3+^ [18]. However, the post-translational regulation of AtSTOP1, in both high and low Pi conditions, remains elusive. As part of the local response, the multicopper oxidase LOW PHOSPHATE ROOT 1 (AtLPR1) changes the iron oxidation state from Fe^2+^ to Fe^3+^ in the root tip apoplast under low Pi conditions, this phenomenon correlates with callose accumulation and the process of differentiation of the root apical meristem (RAM) [19]. Iron redistribution and redox fluctuations, coupled to reactive oxygen species (ROS) signaling, promote the expression of the *CLAVATA3/ENDOSPERM SURROUNDING REGION 14* (*AtCLE14*) gene. AtCLE14 triggers the full differentiation of the RAM via a signaling pathway that PEPTIDE RECEPTOR 2 (AtPEPR2) and CLAVATA 2 (AtCLV2) receptors use, which, in turn, may act negatively upstream of POLTERGEIST (AtPOL) and POLTERGEIST-LIKE 1 (AtPLL1), SCARECROW (AtSCR), and SHORT ROOT (AtSHR) [20,21]. The systemic response to low Pi availability is controlled by the internal concentration of inositol polyphosphate (IPP), and it is mainly modulated by PHOSPHATE STARVATION RESPONSE 1 (AtPHR1), which is a MYB-CC TF that activates the expression of several genes involved in the transport, scavenging, and recycling of Pi [22,23]. This TF was first characterized in *Chlamydomonas reinhardtii* under the name of PHOSPHORUS STARVATION RESPONSE 1 (CrPSR1) [24,25]. In *A. thaliana*, PHR1 controls the transcription of high-affinity Pi transporters to improve nutrient assimilation under Pi deprived conditions. For example, AtPHR1 promotes the expression of *PHOSPHATE TRANSPORTER 1* (*AtPHT1*) to enhance Pi transport across the plasma membrane (PM) [26,27]. AtPHR1 induces the expression of *AtPHT2, AtPHT4, AtPHT3, AtVPT1*, and *AtVPE1* genes, all encoding transporters involved in intracellular Pi redistribution [28,29,30,31,32,33,34,35]. Another aspect of the systemic response controlled by AtPHR1 is the recycling of internal Pi from phospholipids and nucleic acids [36,37]. Pi-deprived plants re-organize the PM composition, replacing membrane phospholipids with non-phosphorus lipids such as sulfoquinovosyldiacylglycerol and digalactosyldiacylglycerol [36,37,38]. Under low Pi conditions, AtPHR1 promotes the transcription of genes involved in lipid turnover such as *AtSQD1* and *AtPLDz2* [37,38]. AtPHR1 also induces *PURPLE ACID PHOSPHATASES* (*AtPAPs*) and *RIBONUCLEASES LIKE 1* (*AtRNS1*) transcription, which are the encoding enzymes that are essential to release Pi from the organic pool [39,40]. AtPHR1 activity is negatively regulated by proteins containing the SIG1-PHO81-XPR1 (SPX) domain (encoded by *AtSPX1*, *AtSPX2*, *AtSPX3*, and *AtSPX4* genes). The post-translational regulation of AtPHR1 by SPX proteins relies on inositol polyphosphate (IPP) levels [23,41,42,43,44]. In high Pi conditions, AtSPX4 binds AtPHR1, retaining it at the cytoplasm [43,44]. At low Pi conditions, AtPHR1 promotes the expression of *AtSPX1/2*, which is an encoding protein that acts as a repressor of AtPHR1 in the nucleus [23,41,42].

The response of plants to Pi scarcity has been mainly explored using the Arabidopsis root as a model. The root is an essential organ for water and nutrient uptake in diverse clades of the Viridiplantae kingdom; however, we should consider that the root is an innovation during land plant evolution that occurred millions of years later than the developmental and physiological mechanisms that allowed plants to thrive in soils with limited water and nutrients. Pioneer studies on the response of *Marchatia polymorpha* to Pi limitation described changes in rhizoid abundance, red thallus pigmentation, and the precocious formation of gemmae cups [45,46,47]. Voth and Hamner (1940) [46] suggested that some segments of thallus tips grow well under low Pi conditions, which may be due to a storage of nutrients which occurred previously facing low Pi conditions. Such studies pointed already to the existence of a molecular program in liverworts to deal with Pi scarcity. However, the molecular and physiological mechanisms of Marchantia to adapt to, and develop in, an environment with low Pi levels remain obscure. In an attempt to shed light on the existence and conservation of such adaptive mechanisms in a rootless early divergent land plant, in this study, we describe the morpho-physiological changes, and the underlying transcriptional profile, of *Marchantia polymorpha* in response to low Pi availability.

## 2. Results

### 2.1. Evolutionary Landscape of Pi Starvation-Induced Genes across the Plant Kingdom

**Figure 1:**
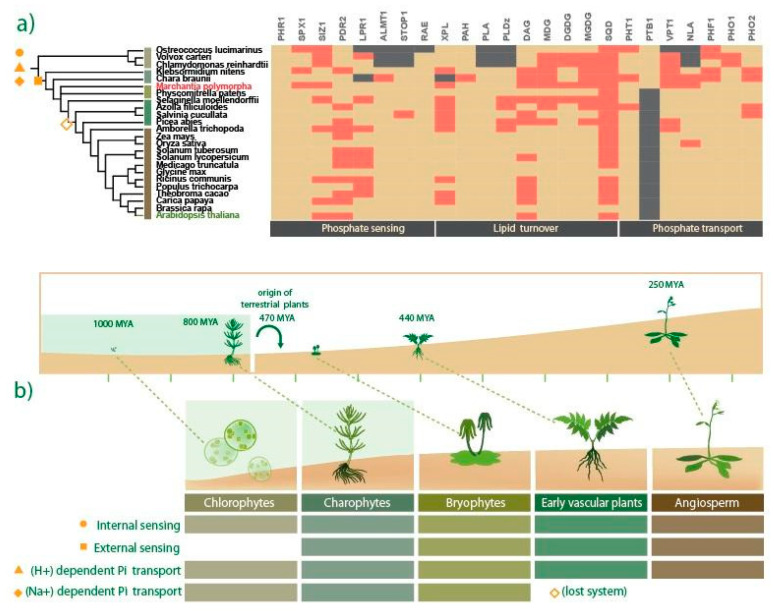
**Evolution of phosphate starvation responses in land plants.** (**a**) Phylogenetic tree containing 24 representative genomes sampled across plant kingdom, the cladogram was taken from the NCBI taxonomy browser (https://www.ncbi.nlm.nih.gov/Taxonomy/CommonTree/wwwcmt.cgi). The heatmap shows a comparative conservation analysis of key genes involved in Pi sensing, lipid turnover, and Pi transport. Red-colored rectangles represent single copy genes, beige-colored rectangles mean that for such proteins there are two or more genes, and gray-colored means that no gene coding for such proteins was identified through our approach (see methods for details). Figures S2–S24 show the phylogenetic reconstruction for each gene analyzed. (**b**) Land plant evolution timeline. A close-up highlight of the morphological differences between chlorophytes, charophytes, bryophytes, gymnosperms, and angiosperms. Color rectangles showed the conservation for key mechanisms to cope with Pi starvation, such as an internal sensing network, external sensing network, (Na^+^) and (H^+^) dependent Pi transporters.

In the last decades, diverse studies revealed how key genetic networks orchestrate adaptive mechanisms of plants to cope with Pi-limited conditions [7,8]. However, little is known about how these strategies evolved along the early divergent lineages of the plant kingdom. To explore the conservation and divergence of such genetic mechanisms, we performed a survey in key representative genomes distributed across plant phylogeny (Figure 1a). Putative homologs were searched by sequence homology and phylogenetic reconstructions, which allowed us to identify the evolutionary history of genes related to Pi sensing, organic acid exudation, lipid metabolism, and Pi transport (Figure 1a). In the Pi sensing category, putative homologs encoding the internal Pi sensing module *PHR1-SPX1* were found in all clades analyzed, from Chlorophyte algae to land plants (Figure 1b). In contrast, in the local Pi sensing genetic network composed of the *STOP1*, *ALMT1*, *LPR1*, *PDR2*, and *RAE1* genes, some members were absent in the Chlorophyte lineage, but each of them was present in all the Streptophyte lineage (Figure 1b). Interestingly, the genome of *Ostreococcus lucimarinus* lacks four of the five genes related to local sensing, but the other Chlorophyte genomes analyzed (*Volvox carteri* and *Chlamydomonas reinhardtii*) possess putative homologs genes of *PDR2*, *LPR1*, and *RAE1* (Figure 1a). The absence of the STOP1–ALMT1 module in the Chlorophyte sampled genomes was evident, whereas in charophyte algae (*Chara Braunii* and *Klebsormidium nitens*), putative homologs of STOP1 and ALMT1 were present (Figure 1a). Our evolutionary analyses highlighted the STOP1–ALMT1 module as a streptophyte innovation. On the other hand, the internal Pi sensing module PHR1–SPX1 was present since early green algae lineages (Figure 1b). Moreover, the putative homologs implicated in lipid turnover, such as XPL, DAG, MGD, DGDG, MGDG, and *SQD* were highly conserved in all the lineages investigated (Figure 1a). In addition, we explored Pi transport mechanisms and found putative homologs of *PHT1* and *PHF1* in all the species analyzed (Figure 1a), but the putative homologs of *NLA* were only present in the streptophyte lineage (Figure 1a). We found that *PTB* genes were present exclusively in both green algae lineages (Chlorophyte and Charophyte) and bryophyte (Figure 1b), as previously reported [48]. Moreover, we found homologs of *VPT* in all sampled genomes, with the exception of *O. lucimarinus* (Figure 1a). The last transporter family analyzed was the AtPHO1, and we found putative homologs in all species analyzed in this study (Figure 1a).

### 2.2. M. polymorpha Exhibits Low Genetic Redundancy in Key Pi-Responsive Regulatory Networks

*M. polymorpha* provides a useful model system due to its well-characterized morphology, anatomy complexity [49], and its low genetic redundancy [50]. Our presence–absence analysis of putative homologs related to PSR (Figure 1a) led us to uncover a low genetic redundancy for key genetic networks in *M. polymorpha*, as compared to other land plants (red rectangles in Figure 1a). For example, the internal Pi sensing module PHR1-SPX1 contains 11 and four members of each family in *A. thaliana*, respectively. In the *M. polymorpha*, we identified only three genes encoding putative PHR/PSR-related MYB-CC TFs (Figure 1a). Our phylogenetic reconstruction placed MpPHR1 (Mapoly0003s0147) and MpPHR2 (Mapoly0098s0044) close to AtPHR6, in contrast to MpPHR3 (Mapoly0115s0048) resides in another subclade with AtPHR8/9/12 (Figure S3). *AtPHR1* and *AtPHL1* form an independent clade that includes protein sequences from the early vascular plant *S. moellendorffii* up to *A. thaliana*, without bryophyte or green algae sequences. A single gene encoding an SPX-class 1 member was found in *M. polymorpha*; Mp*SPX* (Mapoly0002s0123) is phylogenetically closest to AtSPX4 (Figure S2). In the lipid turnover category, there are two *AtDAG* genes, two *AtPAH* genes, two *AtSQD* genes, two *AtDGDG* genes, three *AtMGDG* genes, three *AtMGD* genes, and two *AtPLDz* genes in the *A. thaliana* genome. In the *M. polymorpha* genome, our analysis retrieved single homologs for *PAH*, *MGD*, *DGDG, MGDG*, and *SQD*, (Figure 1a). In the opposite sense, we found nine homologs for *PLDz* and seven for *PLA*. Our phylogenetic reconstructions showed that Mp*PAH* (Mapoly0089s0039), Mp*DAG* (Mapoly0022s0098), Mp*MGD* (Mapoly0023s0061), Mp*MGDG* (Mapoly0023s0061), and Mp*SQD* (Mapoly0010s0049) sequences were in a clade with putative orthologs from *P. patens* and *S. moellendorffii* (Figures S11, S14, S15, S17 and S18). The phylogenetic tree of DGDG family consists of two clades: one for the *AtDGDG1* and the other for *AtDGDG2*, the latter includes the single Mp*DGDG* sequence (Figure S16). Moreover, we found a single vacuolar Pi transporter in *M. polymorpha*, Mp*VPT* (Mapoly0094s0020), the *A. thaliana* genome has three loci encoding for VPTs [35]. Phylogenetic reconstruction of *VPT-like* proteins showed that Mp*VPT* (Mapoly0094s0020) is in a clade with putative homologs from other bryophytes (Figure S20). Finally, the *M. polymorpha* genome has a single homolog, Mp*NLA* (Mapoly0044s0127), and *A. thaliana* possesses two copies where the phylogenetic reconstruction places Mp*NLA* close to other bryophyte sequences (Figure S21).

### 2.3. Phenotypical and Physiological Impacts of Pi Availability on Thallus Development

**Figure 2:**
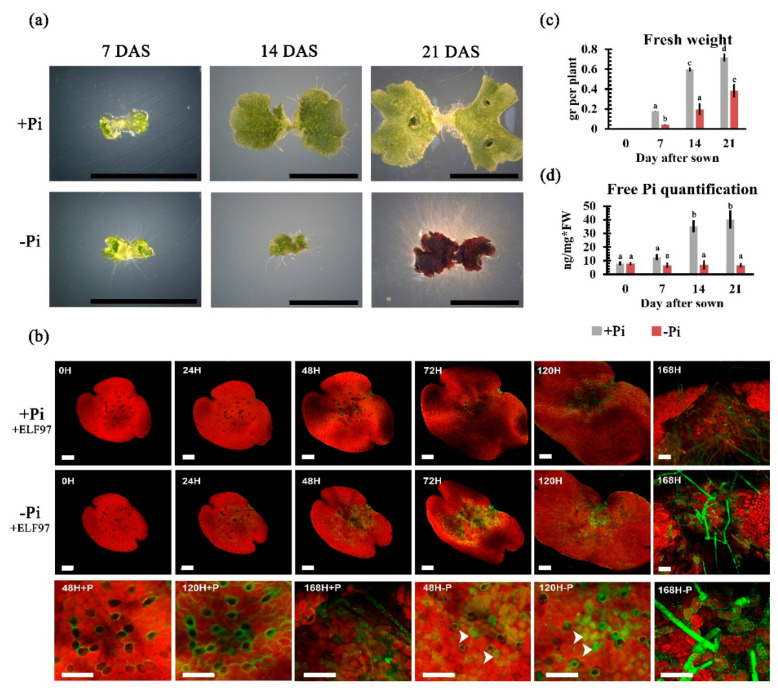
**Phenotypical and physiological effect of phosphate availability in *Marchantia polymorpha***. (**a**) Morphological effect of +Pi and −Pi status on thalli development in plants at 7, 14, and 21 days after sowing (DAS) grown under continuous stressful conditions (black lines = 5 mm). (**b**) In vivo analysis of acid phosphatase activity with ELF-97 in gemmae after 0, 24, 48, 72, and 120 h after sowing. (**c**) Fresh weight (*N* = 30 plants) and (**d**) Free Pi quantification of plants exposed to high and low Pi conditions after 7, 14, and 21 DAS (*N* = 30 plants). The statistical significance was determined with Tukey’s honestly significant difference test, letters show significant differences with a *p* value < 0.01. All bars in confocal images, panel (**b**) =100 μm.

In natural ecosystems, fluctuations of Pi concentrations in the soil drive fine-tuned mechanisms that allow plants to cope with limiting Pi conditions [7,8]. For example, root morphological modifications and physiological adaptations that respond locally to Pi availability have been associated with enhanced Pi assimilation [51]. However, little is known about the phenotypic plasticity upon Pi starvation in plants without roots, especially in liverworts such as *M. polymorpha*. To characterize the morpho-physiological responses to low Pi availability during thalli development, gemmae were grown on agar media supplemented with different concentrations of Pi (0, 10, 25, 50, and 500 µM), and their phenotypes were recorded at multiple time points (7, 14, and 21 days) (Figure S25), allowing us to determine Pi concentrations for high (+Pi) and low (−Pi) growth conditions. The production of a red pigment, most probably auronidin accumulation [52,53], and changes in rhizoid development were observed at 0, 10, and 25 µM of Pi (Figure S25). Thus, taking into consideration that thallus development was severely affected at 0 µM and 25 µM was the highest Pi concentration that induced phenotypic changes, we selected 10 µM as a low Pi availability condition. We defined 500 µM of Pi as the high availability condition, since at this concentration, thalli neither showed an accumulation of auronidin nor differences in rhizoid development (Figure S25). After registering the alteration on rhizoid development under -Pi conditions, we hypothesized that *M. polymorpha* rhizoids may display similar alterations to those described for *A. thaliana* root hairs under Pi starvation. In order to test such hypotheses, we monitored rhizoid development at each time point of the experiment. Our results showed that at 7 days after sowing (DAS), gemmae developed into young thalli in both +Pi and -Pi conditions (Figure 2a). At this stage, we already observed differences in the length of rhizoids under -Pi when compared to +Pi (Figure 2a). The differences between +Pi and -Pi conditions were dramatic in both rhizoid length and density at 21 DAS (Figure 2a). In *A. thaliana*, upon Pi starvation, the increase in root hair density correlates with the excretion of Purple Acid Phosphatases (PAPs) to solubilize Pi from organic compounds [54]. We explored if *M. polymorpha* rhizoids excrete PAPs by using the phosphatase’s substrate EFL-97 or 2-(2′-phosphoryloxyphenyl)-4(3H)-quinazolinone [55], which is converted to 2-(2′-hydroxyphenyl)-4(3H)-quinazolinone, a fluorescent product that emits a green signal at 530 nm wavelength. For this experiment, gemmae were grown under -Pi and + Pi in solid media, both supplemented with 25 µM of ELF-97 (see methods for details). After transference to media and being poured into micro chambers to follow each analyzed gemmae, we recorded confocal sections at 0, 24, 48, 72, 120, and 168 h after sown (HAS). Our results are shown in Figure 2b, where the green signal corresponds to PAP enzymatic activity on ELF-97, and the red signal corresponds to chlorophyll autofluorescence. At 0 HAS, no evident green signal was observed among treatments. By 24 HAS, a discrete green signal was observed in both +P and -Pi conditions. However, at 48 HAS, a stronger green signal was observed in gemmae grown in -Pi, not only in rhizoid cells but also in surrounding cells (Figure 2b magnifications). At this time point, +Pi grown gemmae also showed green signal in the rhizoids; however, the green fluorescence intensity in these cells was lower, and no evident green signal colocalizing with red signal was observed (Figure 2b magnifications). The green signal pattern was even more intense at 72 and 120 HAS under -Pi conditions when compared to those observed in gemmae grown under +Pi conditions at the same time points (Figure 2b magnifications). We also observed in -Pi grown plants that several chlorophyll-producing cells, in the region of the thallus, where rhizoids are formed, also showed a more intense green signal when compared to the control (Figure 2b magnifications). Our phenotypic analysis revealed that the proper development and growth of plants, grown under low Pi conditions, was also drastically altered when compared to those of plants grown in +Pi (Figure 2a). The biomass production was affected lightly after 7 DAS and was clearly lower at 14 and 21 DAS under -Pi conditions relative to the control (Figure 2c). In addition, we observed an evident change in the green color characteristic of the thallus to a strong red/purple color, suggesting that an accumulation of phenylpropanoids (e.g., aunoridins) in response to low Pi conditions is a conserved feature of land plants in response to scarce Pi environments (Figure 2a). The previously described phenotypes correlated with a significant decrease in free Pi in plants grown under low Pi conditions, in comparison with the control (Figure 2d). Collectively, our phenotypic analyses showed that Pi deficiency causes alterations in early thallus development, some of which were analogous to those previously described for *A. thaliana* and other land plant species, suggesting that several phenotypic and physiological responses triggered by this stress are conserved across streptophytes.

### 2.4. Transcriptional Dynamics in Response to Pi Availability

**Figure 3:**
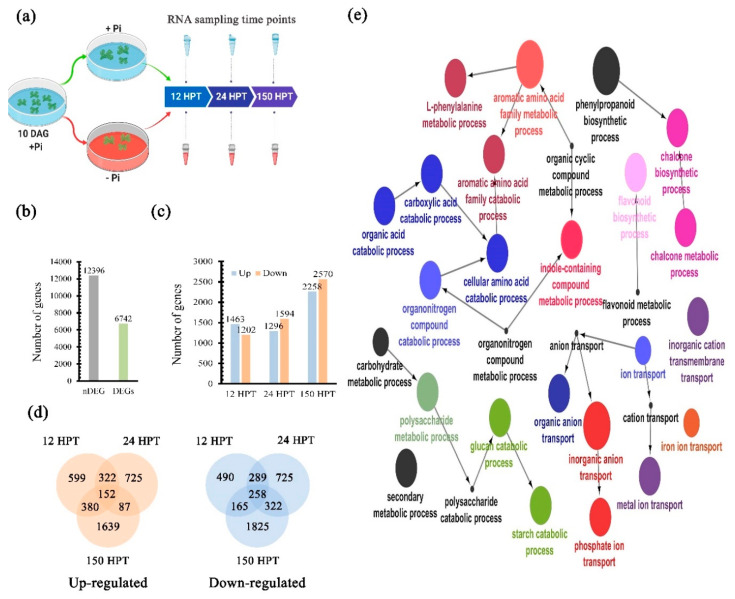
**Dynamics of transcriptional responses to Pi starvation.** (**a**) Graphic description of the experimental design for the RNA-seq approach. (**b**) Differentially expressed genes (DEGs) and non-differentially expressed genes (nDEGs). (**c**) Bar plot showed the up-regulated genes in light red and the down-regulated ones in green for each time of the transcriptome. (**d**) Venn diagrams for up-regulated (Red) and down-regulated (green) genes for each condition. (**e**) Clustering of over-represented Gene Ontology (GO) categories in the genes up-regulated in response to low Pi availability, color clustered similar categories and circle size correlates with the *p*-value of enrichment test.

The transcriptional response upon Pi starvation has been characterized in diverse angiosperms, such as *A. thaliana, Oriza sativa, Zea mays*, and other angiosperms, where the transcriptional responses induced by low Pi availability are broadly conserved [56,57,58,59]. However, the transcriptional response to low Pi has not been explored in earlier diverging lineages of land plants, such as liverworts. To gain insight into the transcriptional program of young plants relative to Pi availability, we employed an RNA-seq strategy in thalli derived from gemmae 10 DAS (Figure 3a). Plants were grown previously on Pi-sufficient liquid media for 10 days and subsequently transferred to either high (500 µM) or low Pi (10 µM) MS liquid media (Figure 3a). We defined the times for tissue collection and RNA isolation at 12, 24, and 150 h post transference (HPT) (Figure 3a), based on the following criteria: (1) the induction of phosphatase activity in low Pi conditions observed at 24 h after transfer, suggesting early transcriptional activation (Figure 2b); (2) the first distinct phenotypic changes, e.g., rhizoid phosphatase activity, were observed at 168 h (7 d) after sowing (Figure 2b); (3) the morphological changes in the number and length of rhizoid cells were qualitative evident at 168 h (7 d) after sowing (Figure 2a and Figure S25); and (4) the acidification of media, probably by the exudation of organic acids and proton extrusion was observed at 7 DAS in Pi limited conditions (Figure S26). In addition, biological replicates were obtained in an independent experiment under the same growth conditions and RNA collection. Our transcriptome resulted in a total of 274,795,583 reads, after removing poor quality reads and adapters. To estimate the gene expression profile, we mapped the reads using the DNA subway platform from cyverse (https://dnasubway.cyverse.org/) against the reference genome V3.1 [50]. To determine differentially expressed genes (DEGs), we contrasted libraries from the low Pi conditions against those from plants grown under high Pi conditions. We applied a fold change of [−1.5 < FC > +1.5] and a q-value < 0.001 as a selection criterion. From a total of 19,138 genes, 6742 (35.22%) were differentially expressed (green bar in Figure 3b), while 12,396 genes did not show significant differential expression (gray bar in Figure 3b). Then, we classified the total DEGs that were up or downregulated at each specific time point (12, 24, and 150 HPT). The bar plot in Figure 3c in orange shows 1463, 1594, and 2258 up-regulated genes. In blue, we found 1202, 1604, and 2570 down-regulated genes at 12, 24, and 150 HPT, respectively. In order to define time-specific and shared DEGs, we performed a Venn diagram analysis for up (orange) and down-regulated genes (blue) (Figure 3d). The up-regulated time-specific DEGs were 599, 625, and 1639 genes at 12, 24, and 150 HPT, and the down-regulated time-specific DEGs were 490, 725, and 1825 at 12, 24, and 150 HPT, respectively (Figure 3d). In addition, 152 up-regulated genes were shared by the three time points, and 258 down-regulated genes were shared among all times. The remaining DEGs were shared between two time points. For example, in the up-regulated group, samples from 12 and 24 HPT shared 332 DEGs, 24–150 HPT shared 87 and 12–150 HPT shared 380 genes. On the other hand, the down-regulated group showed 289 DEGs shared between 12 and 24 HPT, 322 between 24 and 150 HPT, and 165 DEGs shared among 12 and 150 HPT (Figure 3d). In order to reveal the putative functions of DEGs in response to Pi deprivation, we performed a Gene Ontology (GO) enrichment analysis. The total universe of up-regulated DEGs was used to define enriched subcategories from the main category *¨Biological Processes¨* (*BP*) with a threshold of *p*-value < 0.001. Then, we used the ClueGO software [60] to cluster the over-represented categories in a network, where the circle size correlates to p-value and gray lines define interactions between subcategories (Figure 3e). The major enriched category is the *Aromatic acid family metabolic process*; it forms a subnetwork including subcategories such as *Indole-containing compound metabolic process*, *Aromatic amino acid family biosynthetic process*, *Cellular amino acid catabolic process*, *Organic acid metabolic process*, and others (Figure 3e). Into these subcategories, we found genes associated with auxin biosynthesis, organic acid cycle, and exudation. Other highly enriched subnetworks include *Polysaccharide catabolic process*, *Starch catabolic process*, *Glucan catabolic process*, and others. Interestingly, the *Ion transport* category formed another subnetwork, which includes *Phosphate ion transport*, *Iron transport*, and other subcategories (Figure 3e). In these subcategories, we found genes involved in Pi and Fe transport, and putative Pi transporter genes such as homologues of *PTB* and *PHT* were induced in response to low Pi availability. Another category was the *Flavonoid biosynthesis* and *Chalcone biosynthetic process*, where genes related to auronidin biosynthesis were found [52]. The transcriptional activation of these genes correlates with the low Pi phenotype observed (dark purple color on thallus Figure 2a). In addition, we found the conservation of some of the typical responses to low Pi availability such as the induction of genes underlying the plant hormone genetic networks, organic acid exudation, iron transport, Pi transport, and cellular responses to Pi starvation (Figure 3e). Then, we searched for enriched functional categories of up-regulated genes for each time point; these results are available in the Tables S1–S3. The 599 DEGs specific for 12 HPT (Figure 3d) showed enrichment in 66 functional categories, which include *Response to hormones*, *Auxin polar transport*, *Response to cytokinin*, and the *Auxin-activated signaling pathway*, among others (Table S1). We found 570 functional categories enriched in the 725 DEGs specific for 24 HPT (Figure 3d). Some of these are related to plant hormones such as *Response to abscisic acid* and *Response to ethylene* (Table S2). In addition, we found categories related to processes previously associated with the Pi-local response including *Meristem development* and *Defense response by callose deposition in cell wall* (Table S2). The 639 specific DEGS of 150 HPT revealed enrichment in 250 functional subcategories, of which some of the most interesting were *Carboxylic acid transport*, *Citrate metabolic process*, *Programmed cell death*, and *Response to aluminum ion* (Table S3). Together, our time-specific GO analysis reveals that the transcriptional response of *M. polymorpha* to cope with Pi starvation is dynamic and involves the up-regulation of genes associated to several key processes, some of which were time-specific and conserved partially with those described in *A. thaliana, O. sativa*, and *Z. mays* [56,57,58,59].

### 2.5. Transcriptional Regulation of the Local and Systemic Pi-Sensing Modules

In order to explore how Pi sensing occurs in *M. polymorpha*, we analyzed the transcriptional behavior of genes involved in local and systemic perception networks. Interestingly, only Mp*PHR1* was up-regulated in low Pi, in contrast to the other two MYB-CC genes (Figure S27). This suggests that Mp*PHR1* is functionally equivalent to *AtPHR1*. Moreover, Mp*SPX* was up-regulated across all time points in response to low Pi, similar to that observed for AtSPX1/2. With respect to the local perception network, we found that Mp*STOP2* was significantly down-regulated at 12 HPT and up-regulated at 24 and 150 HPT, under Pi deficiency. Based on its transcriptional activation, we hypothesized that Mp*STOP2* (Mapoly0083s0069) may be functionally equivalent to *AtSTOP1*. Mp*ALMT3* (Mapoly0100s0061) and Mp*ALMT2* (Mapoly0070s0016) were significantly down-regulated at 12, 24, and 150 HPT, even when Mp*STOP2* was up-regulated (Figure S27). Although we identified 13 loci encoding multicopper oxidases (Mp*LPR1 to* Mp*LPR13*), only two of them, Mp*LPR1* (Mapoly0008s0168) and Mp*LPR3* (Mapoly0008s0270), were up-regulated in all three time points sampled in this work (Figure S27). Mp*LPR4* (Mapoly0030s0116) and Mp*LPR7* (Mapoly0186s0019) were up-regulated at 12 HPT and then down-regulated at 24 and 150 HPT. Mp*LPR11* (Mapoly0217s0007) was up-regulated at 12 and 24 HPT and then down-regulated at 150 HPT (Figure S27). Only Mp*LPR2* (Mapoly0004s0015) was down-regulated in the three time points analyzed (Figure S27). On the other hand, the CLE peptide family in *M. polymorpha* contains two members (Mp*CLE1* and Mp*CLE2*) [61,62]; under low Pi conditions, Mp*CLE1* (Mapoly1011s0001) was down-regulated at 12 and 24 HPT, and Mp*CLE2* (Mapoly0084s0052) was up-regulated at 12, 24, and 150 HPT (Figure S27). Taken together, these results indicate that despite the differences, at morphological and genetic levels, with Arabidopsis, *M. polymorpha* displays a partially conserved transcriptional response of both local and systemic Pi-sensing pathways.

### 2.6. Low Pi Promotes the Induction of Genes Related to Organic Acid Synthesis and Exudation

To unveil if genes involved in organic acid synthesis and exudation were affected in response to low Pi conditions, the transcriptional profiles of key genes such as the malate synthase (MLS), citrate synthase (CIS), malate transporters (ALMT), and citrate transporters (MATE) were analyzed (Figure S28). Our search in the *M. polymorpha* genome with the Pfam identifiers PF00285 for citrate synthase, PF01274 for Malate synthase, and PF01554 for the MATE transporter revealed five loci encoding putative *MALATE SYNTHASE* (Mp*MLS1 to* Mp*MLS5*), from which the transcripts of Mp*MLS2* (Mapoly0016s0095), Mp*MLS3* (Mapoly0023s0108), and Mp*MLS5* (Mapoly0154s0035) were down-regulated under -Pi conditions (Figure S28). On the other hand, we found nine loci annotated as *CITRATE SYNTHASE* (Mp*CIS1 to* Mp*CIS9*); four of them were differentially expressed under low Pi conditions. Mp*CIS1* (Mapoly0063s0010) was up-regulated at 12 HPT and down-regulated at 24 and 150 HPT, Mp*CIS2* (Mapoly0071s0012) and Mp*CIS5* (Mapoly0111s0039) were down-regulated at 12 and 24 HPT, and up-regulated at 150 HPT (Figure S28), and Mp*CIS6* (Mapoly0200s0007) was down-regulated at the three times analyzed (Figure S28). Regarding malate transporters, Mp*ALMT3* and Mp*ALMT4* were down-regulated, as previously mentioned. This observation correlates with reduced levels of the expression of malate synthesis enzymes and suggests a reduced production and exudation of malate under low Pi conditions. In the case of genes related to citrate transport, we identified 15 loci encoding putative MATE-type transporters (MpMATE1 to MpMATE15), (Figure S28). In Arabidopsis, some members of this family of transporters are involved in citrate exudation [63]. Interestingly, Mp*MATE1* (Mapoly0002s0036), Mp*MATE3* (Mapoly0005s0170), and Mp*MATE10* (Mapoly0041s0128) were up-regulated at all the time points sampled (Figure S28). Additionally, another six members of the MATE family were up-regulated in at least one time point sampled (Figure S28). These results suggest that *M. polymorpha* promotes preferentially the synthesis and efflux of citrate rather than malate to cope with low Pi.

### 2.7. Pi Starvation Induces the Expression of Genes Involved in Lipid Turnover

To explore if phospholipid remodeling takes place in *M. polymorpha* in response to Pi limited conditions, as previously reported for *A. thaliana* [36,37,38], we searched for putative orthologs of key enzymes involved in phospholipid degradation and glycolipid synthesis (Figure 1a). Our results revealed seven loci encoding putative orthologs of type D phospholipases (PLDs), and our phylogenetic reconstruction showed that *M. polymorpha* PLDs formed a basal clade with PLD sequences of bryophytes (Figure S13). Among the *M. polymorpha* PLD-encoding loci, we found that Mp*PLD1* (Mapoly0191s0011) is up-regulated at the three time points analyzed and displayed the strongest FC value at 150 HPT (Figure S29). This suggests that Mp*PLD1* is probably the functional equivalent of *AtPLDz2*, which is also highly up-regulated in response to Pi deprivation [37]. Although other members of the *PLD* family, genes in *M. polymorpha* that were down-regulated and up-regulated at different time points showed lower FC values than those of Mp*PLD1* (Figure S29). In the case of enzymes involved in galacto and sulpholipid synthesis, we found single genes encoding them, Mp*PAH*, Mp*DGDG*, Mp*DAG*, Mp*MGDG*, and Mp*SQD*. Among them, Mp*DGDG* and Mp*MGDG* did not show significant changes in their expression relative to the availability of Pi, and Mp*DAG* was down-regulated at 12 and 24 HPT and up-regulated at 150 HPT. In contrast, Mp*SQD* was up-regulated at 12 and 24 HPT and down-regulated at 150 HPT in low Pi (Figure S29). Altogether, these data show that a group of genes, whose homologs in Arabidopsis are involved in lipid turnover, are up-regulated in response to Pi availability in *M. polymorpha*.

### 2.8. Transcriptional Behavior of Genes Involved in ROS Synthesis in Pi-Starved Thallus

Previous studies have associated reactive oxygen species (ROS), such as hydroxyl radical (OH^−^), superoxide (O^−^), and hydrogen peroxide (H_2_O_2_), to the regulation of plant development by acting as secondary messengers [64]. It has been shown in Arabidopsis that NADPH OXIDASE (NOX1) or *RESPIRATORY BURST OXIDASE HOMOLOG (AtRBOH*), a major enzyme in the production of ROS, is required for the appropriate root hair developmental patterning [65,66]. Moreover, transcriptional studies of *A. thaliana* upon Pi starvation revealed that peroxidase-encoding genes were significantly up-regulated [16]. In order to explore the conservation of this response in the early divergent land plant *M. polymorpha* in response to Pi starvation, the transcriptional profiles of *NOX/RBOH* and peroxidase-encoding genes were analyzed. The Pfam identifiers PF08414 (NADPH oxidase) and PF00141 (Peroxidase) were used to identify the putative *M. polymorpha* orthologs. We found two loci for *NADPH OXIDASE* (Mp*NOX1* and Mp*NOX2*) (Figure S30). The expression of Mp*NOX1* (Mapoly0258s0001) was up-regulated at 12 and 150 HPT, but it was down-regulated at 24 HPT (Figure S30), while Mp*NOX2* (Mapoly0046s0097) was down-regulated at the three time points sampled (Figure S30). In contrast, we found 221 loci putatively encoding peroxidases (PRX), from which 100 loci showed differential expression in our transcriptomic approach (Figure S30). Transcripts of 24 loci were constantly down-regulated and 14 were up-regulated in the three time points analyzed, and the remaining 62 were up or down-regulated in at least in one of the time points sampled (Figure S30). The transcriptional induction of Mp*NOX1* under low Pi conditions suggests that *M. polymorpha* promotes the generation of superoxide radical (O^−^). In addition, the strong induction of several Mp*PRX* genes might correlate with ROS production under low Pi conditions and could be involved in the changes observed in rhizoid development upon Pi scarcity.

### 2.9. Validation of Transcriptome by qPCR

**Figure 4:**
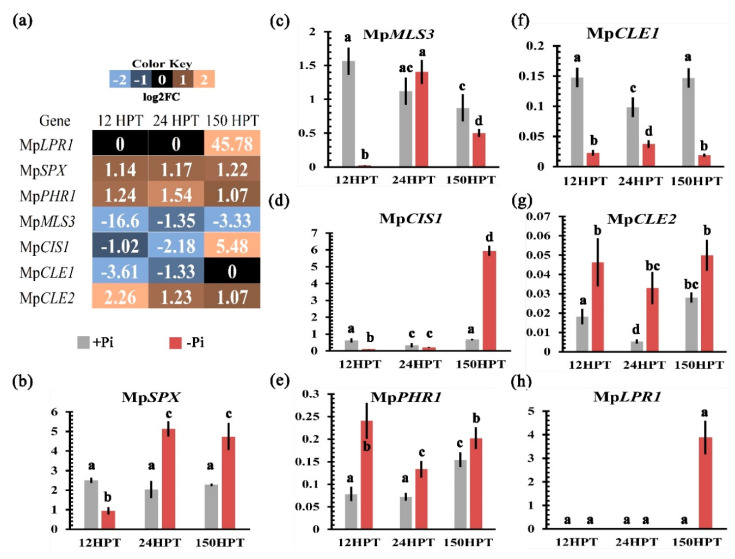
**Validation of transcriptome by quantitative real-time qPCR.** (**a**) Heat map showed the transcriptional profile of Mp*SPX*, Mp*PHR1*, Mp*CLE2*, Mp*LPR1*, Mp*CIS1*, Mp*CLE1*, and Mp*MLS3* resulting from our transcriptome. The gradient of colors represents the following: light blue is down-regulated, black means no change in expression, and orange represents up-regulation. (**b**–**h**) Transcript levels of Mp*SPX*, Mp*PHR1*, Mp*CLE2*, Mp*LPR1*, Mp*CIS1*, Mp*CLE1*, and Mp*MLS* genes at 12, 24, and 150 HPT, as determined by qRT-PCR. The Y-axis for all bar plots represents relative expression. The Tukey HSD test was applied for each set of data to find significant differences with an alpha value of 0.05; the letters in bars show the relative expression differences in the genes analyzed.

In order to evaluate the quality of our transcriptome, seven genes were selected for qPCR analysis. The putative homologs of the selected genes have been associated with relevant aspects of the Pi starvation responses in diverse plant species, such as Pi sensing, cellular response to Pi, organic acid biosynthesis, and transport. Actin was used as control, since it has proven to be an accurate control in previous analyses in diverse stress conditions [67] (Figure 4a). For our qPCR assays, we used cDNA derived from the total RNA sampled at the same time points and growth conditions used for our transcriptome. The transcriptomic profiles of the selected genes are shown in Figure 4a, in which up-regulated genes are depicted in orange, down regulated ones are depicted in blue, and unchanged ones are depicted in black. For the internal Pi perception, our transcriptome showed that Mp*PHR1* and Mp*SPX* were up-regulated in the three time points analyzed under low Pi conditions (Figure 4a). In contrast, our qPCR analysis showed that Mp*SPX* transcripts under low Pi conditions were less abundant at 12 HPT, but they increased drastically at 24 HPT and 150 HPT compared to high Pi conditions as the control (Figure 4b). In the case of Mp*PHR1*, transcript levels increased significantly in response to Pi starvation at the three time points tested (Figure 4e). These qPCR results denote a high coherence with our transcriptomic data for these genes. Regarding organic acid biogenesis, our transcriptomic approach showed that transcript levels of Mp*MLS3* were down-regulated at all time points sampled and those of Mp*CIS1* were down-regulated at early times (12 and 24 HPT) and up-regulated at 150 HPT (Figure 4a). The qPCR analysis revealed that transcript levels of Mp*MLS3* were significantly lower in low Pi at 12 and 150 HPT with respect to the control, but no significant differences between high and low Pi conditions were observed at 24 HPT (Figure 4c). Our qRT-PCR results for Mp*CIS1* showed no significant differences at early times (12 and 24 HPT), but they highly increased at 150 HPT under low Pi conditions (Figure 4d). Finally, we analyzed the transcriptional behavior of genes whose putative orthologs in *A. thaliana* are involved in the local perception of Pi availability. Our transcriptome showed that Mp*CLE1* was down-regulated at early times (12 and 24 HPT), whereas the Mp*CLE2* was up-regulated in the three points sampled, in response to low Pi (Figure 4a). The qPCR data for Mp*CLE1* showed low transcript abundance in -Pi conditions at the three time points tested (Figure 4f); on the other hand, transcript levels for Mp*CLE2* were high under low Pi conditions at the three time points sampled (Figure 4g), showing a high correlation with the expression pattern observed for this gene in our transcriptome approach. In the case of Mp*LPR1*, the transcriptome data showed that the transcription of this gene is not altered between treatments at early times (12 and 24 HPT) and is up-regulated under low Pi at 150 HPT (Figure 4a). Our qPCR analysis corroborated this expression pattern only after 150 HPT *MpLPR1* showed high transcript levels under low Pi conditions (Figure 4h). Overall, our qPCR analysis validated the transcriptional behavior of most of the genes selected, indicating that our transcriptome approach is robust and that the reported expression patterns indeed reflect the response of *M. polymorpha* to low Pi.

### 2.10. Determination of PHR1 Binding Sites (P1BS) Enrichment on DEGs

**Figure 5:**
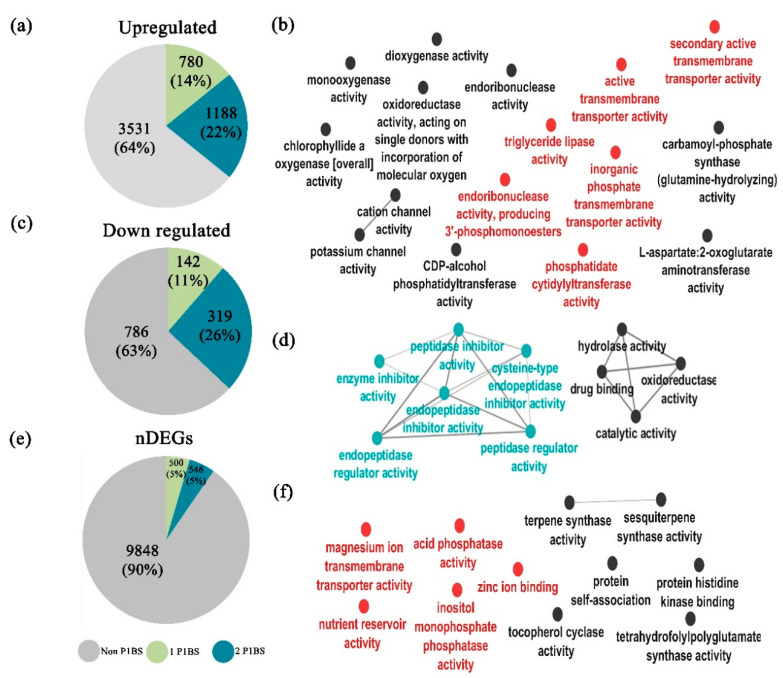
**Genome-wide identification of PHR1 binding sites (P1BS) distribution on *M. polymorpha*.** (**a**) Pie chart showing the number of promoter regions of up-regulated DEGs that lack the P1BS motif (in gray) and those whose promoter has one (green) or two (blue) motifs; the same color code applies for those pie charts y (**c**) and (**e**). (**b**) Illustrates the GO analysis of the up-regulated DEGs that contain the P1BS CREs; red color indicates the subcategories that are strongly associated to Pi starvation responses, and black indicates those terms that have no evident link to the Pi starvation responses (PSR). (**c**) Pie chart showing the number of promoter regions of down-regulated DEGs that lack the P1BS motif and those whose promoter has one or two motifs. (**d**) The GO network for the down-regulated genes with P1BS motifs, blue nodes indicate subcategories that have been related to PHR1 function in regulating genes involved in other stress conditions described in *A. thaliana* [71], dark nodes show categories with no evident link to the Pi starvation response. (**e**) Pie chart showing the number of promoter regions of non-DEGs that lack the P1BS motif and those whose promoter has one or two motifs. (**f**) The GO networks found after analysis of the genes with P1BS motives in the nDEGs group; subcategories related to low Pi availability appear in red, whereas black indicates the subcategories non-associated to the Pi limitation response.

Previous studies in *A. thaliana* reported the enrichment of the PHR1 binding sites (P1BS) *cis* regulatory elements (CREs) in the promoter regions of *AtPHR1* target genes [13,68]. Such genes were transcriptionally impaired in the loss of function mutant *phr1* and the double mutant *phrphl1*, unveiling the relevance of the P1BS in the control of Pi starvation responses [68]. We hypothesized that some of the genes that are differentially expressed in response to Pi starvation in *M. polymorpha* may correlate with the presence of the P1BS motif in their promoter regions. In order to test this idea, we surveyed for the presence and enrichment of the P1BS motif within a 3 Kb region upstream of the transcription start site of DEGs and non-differentially expressed genes (nDEGs). First, we searched the P1BS consensus sequence (*GNATATNC*) in the promoters of the 6746 DEGs using HOMER (detailed in Methods) and found that 3259 of them harbor at least one P1BS. Then, we performed a motif enrichment analysis in the promoters of those 3259 DEGs and in the 10,894 nDEGs using the Analysis of Motif Enrichment (AME, Version 5.1.1) [69] tool from the MEME suite [70]. AME identifies if the motif, in this case P1BS, is enriched in a set of sequences considered as primary (DEG and nDEG promoters) compared to a control set of sequences (the 16240 *M. polymorpha* annotated promoter regions). AME scores every sequence in terms of the presence of P1BS and performs a statistical analysis to determine if the primary sequences have better scores than the control sequences. Our results show that 2429 promoters of DEGs are enriched for P1BS, and nDEGs are enriched for 946. Then, promoters from DEGs were classified as up-regulated and down-regulated. From the total of up-regulated DEGs (5499 promoters), 3531 have no P1BS, 1188 bear two P1BS, and 780 have one P1BS (Figure 5a). Then, we explored the GO categories of the up-regulated transcripts whose promoters have one and two P1BS, and we found several subcategories highly associated with Pi starvation responses (PSR) such as *inorganic phosphate transmembrane transporter activity, triglyceride lipase activity, endoribonuclease activity*, and others colored in red in the network (Figure 5b). The subcategories not related directly to PSR appear colored in black (Figure 5b). In the case of down-regulated DEGs, we analyzed a total of 1247 promoter regions for enrichment in the P1BS CRE and found that 142 have one P1BS, 319 have two P1BS, and 786 have no P1BS (Figure 5c). Respective GO analysis for those that contain the P1BS motif revealed subcategories that are linked to the pathogen response, i.e., *endopeptidase inhibitor type C*, in blue [71], and other subcategories are colored in black (Figure 5d). The last group contained genes whose expression did not show significant changes (10894 nDEGs), and we found that only 500 and 546 promoters have one and two P1BS, respectively (Figure 5e). In contrast, the remaining 9803 have no P1BS. The GO analysis of this group revealed categories that in Arabidopsis have been related to Pi starvation (in red) and stress responses in others that have been previously related to AtPHR1, i.e., *Zinc ion binding and inositol monophosphate phosphatase activity* [71,72] (Figure 5f). Overall, these results show that the number of promoters enriched for P1BS is higher in up-regulated DEGs than in nDEGs. However, the up-regulated and down-regulated promoter groups displayed similar tendencies for P1BS enrichment, but the GO analysis revealed that within the up-regulated group, we mainly found subcategories related to PSR. By contrast, in the down-regulated targets, we found an overrepresentation of subcategories related to the response to pathogens. Hence, the presence of P1BS in the down-regulated and nDEGs suggests that MpPHR1 could be involved in the transcriptional regulation of diverse targets involved in a broad range of stress conditions [71,72,73].

## 3. Discussion

### 3.1. Morpho-Physiological Responses upon Pi Starvation

**Figure 6:**
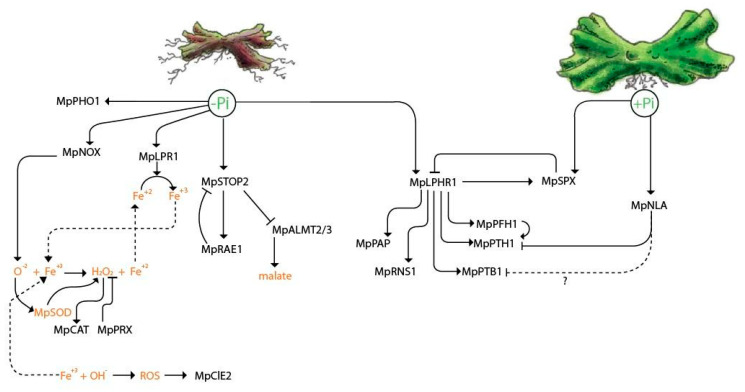
**Model of the molecular response of *M. polymorpha* upon Pi starvation.** Under low Pi conditions (left side of the figure), the expression of *MpPHO1* is promoted. Mp*NOX1* induces the production of superoxide radical (O^2−^), which is catalyzed by SUPEROXIDE DISMUTASE (Mp*SOD*) into hydrogen peroxide (H_2_O_2_). Hydrogen peroxide is catalyzed by CATALASE (Mp*CAT*) into oxygen and water. In addition, the peroxidases (Mp*PRX*) use hydrogen peroxide to carry out several reactions. On the other hand, the (O^2−^) plus Fe^3+^ carried out the Fenton–Weiss reaction to produce hydroxyl radical and Fe^2+^. This step coupled to the induction of Mp*LPR1*, which is a multicopper oxidase that leads to Fe^3+^ from Fe^2+^ under low Pi conditions, generates an oxidation–reduction process, and probably impacts thallus development similar to what occurs in *A. thaliana* root. Here, we observed that the gene encoding the Mp*CLE2* peptide is up-regulated in specific times after growth in low Pi conditions—probably to modulate the maintenance and function of the meristem. In addition, during low Pi deficiency, the expression of Mp*STOP2* is up-regulated, promoting the expression of its putative negative regulator the F-Box ubiquitin ligase Mp*RAE1*. Contrary to previous reports in vascular plants, Mp*ALMT2/3* are down-regulated under low Pi conditions. In agreement with this, we hypothesized that low Pi represses the expression of malate transporters to reduce the efflux on soil. On the other hand, the transcription factor Mp*PHR1* is induced under low Pi conditions and promotes the expression of MpPAPs and Mp*RNS1*, which are both genes involved in Pi scavenging. *MpPHR1* promotes the expression of both Pi transporters MpPHTs and MpPTBs via the P1BS motives in their promoters. Additionally, we found that the Mp*PHF1*, an essential protein for the movement of MpPHT transporters from the ER to the PM, is under the control of Mp*PHR1*. In contrast, Mp*NLA is* expressed in high Pi conditions, leading to the degradation of PHT and probably the degradation of the MpPTB transporters. Finally, among the Mp*PHR1* targets induced during Pi limitation, we found Mp*SPX*, which is a negative regulator of Mp*PHR1*. MpSPX is up-regulated under low Pi conditions under the control of MpPHR1, and another mechanism leads to its expression on high Pi availability.

In the present work, the morphological and physiological responses of the rootless plant *M. polymorpha* to low Pi availability were described. Among these responses, we found an increase in the size and number of rhizoids under low Pi conditions (Figure 6). In *A. thaliana*, the increase in root hair density during Pi deprivation has been described as a strategy to improve the assimilation zone in the rhizosphere [51]. Hence, the modulation of rhizoid development under limited conditions of Pi could be involved in enhancing Pi uptake. It has been shown in *M. polymorpha* that rhizoid development is regulated by auxin signaling and metabolism [73,74,75,76]. We also found that a biomass accumulation of thalli was negatively affected under low Pi conditions, as shown in Figure 6. This phenotypic change, as a function of Pi availability, has been also observed in other plant models such as in *Arabidopsis thaliana*, *Oryza sativa*, and *Zea mays* [35,43,58]. We also demonstrate that free Pi levels are lower in plants grown under -Pi conditions compared to those grown in +Pi conditions, suggesting that the decrease of internal Pi amounts in *M. polymorpha* may trigger the expression of MpPHR1 targets, as reported in *A. thaliana* (Figure 6). In *A. thaliana*, the transcriptional activation of *AtPHR1*, under low Pi conditions, is controlled by AtARF7 and AtARF19 through three auxin response elements found in its promoter [77], suggesting that MpARFs may regulate Mp*PHR1*. However, future experiments are necessary to test this hypothesis. After 21 days of growth under low Pi, thalli exhibited an intense red/purple color, denoting an accumulation of phenylpropanoids (i.e., auronidins in the case of *M. polymorpha*), which is a common phenotype associated with Pi deficiency across land plants. In *A. thaliana*, the activity of key genes such as the *PHENYL AMONIOLIASE* (*AtPAL*) and *CHALCONE SYNTHASE* (*AtCHS*), which are both involved in anthocyanin biosynthesis, were up-regulated under low Pi conditions [56]. Our GO analysis of up-regulated DEGs revealed enriched functional categories related to *Flavonoid biosynthesis* and *Chalcone metabolic processes*, which are both related to auronodin biosynthesis. The biochemical features of auronidin have been reported early, as well as the effects of UV light [78] and nutrient deprivation [52,79] in triggering its synthesis [53]. Here, we report changes in the transcriptional patterns on key biosynthetic genes such as chalcone synthase, phenyl amonieliase, and the transcription factor Mp*MYB14* (Mapoly0073s0038) mainly at 150 HPT (Figure S31). Another important aspect of the Pi-starvation response reported in vascular plants is the secretion of PAPs and OA to the rhizosphere. Our in vivo analysis of phosphatase activity revealed a strong induction of PAP´s activity in response to limited Pi, as previously reported for *Z. mays*, *O. sativa*, and *C. reinhardtii* [71,72,73,74,75,76,77,78,79,80,81,82]. Our results suggest a partial conservation in *M. polymorpha* in the induced OA production, while the transcriptional evidence suggests a preference in the synthesis and exudation of citrate rather than malate.

### 3.2. Evolutionary Perspective of Pi Starvation Responses along Viridiplantae

Pi sensing capacity is one of the major tools of plants to cope with Pi scarcity. In *A. thaliana*, two independent networks involved in the perception of internal and external Pi levels have been described. Our evolutionary analysis unveiled a high conservation of the PHR1–SPX1 module for the internal Pi sensing networks in *M. polymorpha*, and we found that putative homologs have been conserved since chlorophyte algae (Figure 1a; Figure 6). However, the functional conservation of the PHR1–SPX module in green algae and *M. polymorpha* remains pendant. On the other hand, we found that the STOP1–ALMT1 regulatory network is a key innovation of streptophyte lineages (Figure 1a; Figure 6). Therefore, we speculate that the STOP1–ALMT1 network was an innovation that influenced OA efflux and modified earth crust during the silicate weathering period, perhaps helping to reduce the levels of atmospheric CO_2_ [83]. The replacement of phospholipids by glycolipids in the lipidic bilayer of cell membranes is a well-described mechanism to deal with Pi scarcity, which is highly conserved, even in bacteria [84]. We found a strong conservation of *XPL*, *PHA*, *DAG*, *MDG*, *DGDG*, *MGDG*, and *SQD* putative orthologous genes from chlorophyte algae, streptophyte algae, and *M. polymorpha*. However, genes encoding *PHOSPHOLIPASE alpha* (*PLA*) and *PHOSPHOLIPASE D* (*PLD*) were not found in the chlorophyte genomes analyzed. We hypothesized that other phospholipases, such as *PHOSPHOLIPASE C* (*PLC-L*) and *PHOSPHOLIPASE B* (*PLB*), could be converting phospholipids into DAG for the production of sulfo and galactolipids, as it was reported for *C. reinhardtii* under nitrogen limitation [85]. Other key genes that we found to be highly conserved are the putative PHT1 transporters, which are found along plant phylogeny from chlorophyte algae to vascular plants (Figure 1a). However, we found that sodium-dependent Pi transporters (PTB) are present only in green algae and *M. polymorpha*, suggesting that vascular plants lost this type of transporters. In agreement with these findings, the conservation of PTB transporters in *M. polymorpha* was reported in a previous study [48]. In addition, *VPT* and *PHO1* orthologous genes were found highly conserved from green algae to land plants. *NLA* and *PHF* genes, involved in the regulation of PHT transporters, were present only in the streptophyte lineages (Figure 1a). The establishment of this regulatory mechanism could be linked to the process of land colonization by plants as a possible innovation in the regulation of Pi uptake in terrestrial environments. Probably, this innovation occurred to avoid the Pi toxicity in soil, since the concentration of available Pi on land is higher than in aquatic environments.

### 3.3. Transcriptional Response to Cope with Limited Pi in M. polymorpha

Our work opens several working hypotheses, which include one very interesting gene: *MpNOX1*, which is partially up-regulated under low Pi conditions, suggesting that Pi limitation promotes superoxide radical production via *MpNOX1*, connecting ROS signaling to morphological responses (Figure 6), probably to modulate rhizoid development, similarly to what occurs in *A. thaliana* root hairs [65]. Other interesting genes identified were those encoding CLE peptides; the expression of Mp*CLE2* was up-regulated, whereas Mp*CLE1* was down-regulated under low Pi (Figure 4; Figure 6). Previous work showed that the ectopic expression of Mp*CLE1* reduces the thallus growth area of Marchantia plants grown in sufficient Pi conditions; on the other hand, the loss of function *mpcle1* mutant [61] alters the thallus proliferative region and affects the organization of dividing cells. On the other hand, exogenous application of the MpCLE2 peptide alters the differentiation of stem cell-derived daughters, causing the accumulation of the stem cell pool which, after MpCLE2 removal, leads to dichotomous branching [62]. Our transcriptional analysis showed that Mp*CLE2* levels are induced upon Pi starvation, and those of Mp*CLE1* are repressed. We hypothesized that under low Pi conditions, the altered expression of both Mp*CLE1* and Mp*CLE2* may be affecting the proper organization and maintenance of the meristem, which could be reflected in the aberrant development of the thalli observed in plants growing for prolonged time under Pi conditions. Future studies including time-lapse experiments of reporter lines for both Mp*CLE* genes will be very useful to explore this hypothesis.

Our study describes the transcriptional response of *M. polymorpha* upon Pi starvation and integrates a genome-wide search for the P1BS motive enrichment. Our results show a clear enrichment in P1BS motifs in promoters of DEGs as compared to the nDEGs universe (Figure 5a,c,e). The similar enrichment observed in both up-regulated and down-regulated genes suggest that MpPHR1 or other MYB-CC TFs could participate in modulating the expression of genes involved in other processes. This hypothesis is in agreement with early reports in *A. thaliana*, where AtPHR1 was shown to be involved in the regulation of genes related to zinc and iron homeostasis [71,73]. Moreover, AtPHR1 controls the expression of some genes involved in the plant immune response, indicating that the genetic network controlled by AtPHR1 is highly complex and responds to several environmental cues [71]. Despite the number of up-regulated DEGs being higher than that of down-regulated DEGs, we speculate on why a gene that has the P1BS motif in its promoter is down-regulated in response to Pi starvation. One possibility is that if we consider that MpPHR1 and MpPHR-like TFs are all able to bind the P1BS motif, some of the members of the family may act as repressors over a subset of targets. This type of opposite action of diverse members of a TF family has been shown in *M. polymorpha* for MpARFs [75,86]. Future studies should explore such a scenario, since the GO analyses of the down-regulated DEGs, containing the P1BS, revealed interesting functional categories such as *Cysteine type endopeptidase inhibitor*, which contain genes in which the homologs in other species are expressed in response to diverse plant pathogen and some components of the plant immune system [71,87]. This suggests that *M. polymorpha* might also be prioritizing the nutrition mechanisms over the pathogen defense response, as it occurs in other plant species. Such defense plasticity could allow the interaction with beneficial organisms [71]. Future studies should include the generation loss of function mutants of Mp*PHR1* and *MpPHR-like* genes in order to know the direct targets for each of them, as well as to explore their regulatory roles in response not only to low Pi but also upon other biotic and abiotic stresses. Collectively, our morphophysiological, transcriptional, and promoter analyses for P1BS revealed a core of partially conserved responses deployed to cope with low Pi availability in *M. polymorpha*, which were analogous to those displayed by *A. thaliana*. We consider that such conserved adaptations, and the *M. polymorpha* specific traits, should be revised and experimentally tested, case by case, in order to characterize in detail the global strategy of this liverwort to deal with Pi scarce environments.

## 4. Materials and Methods

### 4.1. Evolutionary Analysis

In this work, we searched for classical genes related to phosphate sensing, root system architecture modification, organic acid exudation, and phospholipid turnover along the viridiplantae phylogeny. We selected, as the query, previously characterized Arabidopsis genes involved in the response [7,8]. To identify the orthologous genes in *M. polymorpha* and other 23 representative genomes along the Viridiplantae phylogeny (Figure S1), we performed a search by sequence homology with the software blast v.3.1 [88] used as a threshold parameter an e-value 1e-30 for land plants. For green algae genomes, we used an e-value 1e-10. Additionally, reciprocal blasts against the Arabidopsis protein database TAIR.ver.10 under the same threshold conditions were carried out. The multiple sequence alignment (MSA) was generated with MAFFT software version 7, on the online website [89]. Afterwards, the MSA was trimmed using TrimAL software version 3, maintaining at least 40% for each original protein alignment [90]. Then, we selected the best model fit to carry out the phylogenetic inference. The ModelTest software was used to analyze the sequences according to ACR criteria [91]. For the phylogenetic reconstruction, we used RAxML software on the CIPRES environment (https://www.phylo.org/portal2/home.action) [92]. The bootstrapping value used for each reconstruction was 1000, and the best tree was plotted using iTOL (https://itol.embl.de/) [93].

### 4.2. Plant Material and Growth Conditions

Gemmaelings from *M. polymorpha* (Tak-1) were sown under Murashige and Skoog (MS) media (1/10), pH 5.5, 1% of sucrose (*w/v*) and agar 10 g/L. Plants were grown in Percival chambers with continuous light, with a temperature of 22 °C and humidity around to 60%. To determine the Pi concentration for high and low availability, gemmaelings were exposed to a gradient of K2PO4 (0, 10, 25, 50, 100 and 500 µM) for 21 days. Phenotypic effects were followed and photographed, using a Zeiss stereoscope, at 7, 14, and 21 days after sown (DAS). For further experiments, we used for low Pi (−Pi) a 10 µM concentration and for high Pi (+Pi) a 500 µM concentration of K2PO4. In addition, for RNAseq experiments, we use MS (1/10) liquid media, pH 5.5, 1% of Sucrose (*w/v*), supplemented with K2PO4 to establish the +Pi and -Pi conditions. The implementation of liquid media for RNAseq sample preparation was due to in solid media thallus-carried agar traces in the rhizoids. The plants grow in +Pi conditions for 10 days under continuous light, 22 °C, and humidity around to 60%. Then, we washed the thallus with distilled water and transferred it to +Pi and -Pi media. After 12, 24, and 150 h, the tissue was collected for RNA extraction.

### 4.3. In Vivo Phosphatase Activity Assay

The gemmae from *M. polymorpha* (Tak1) were sown under MS media (1/10) supplemented with 1% of sucrose (*w/v*), phytagel 3 g/L, pH 5.5 and displayed on polycarbonate cell growth chambers. In addition, the Pi (KH2PO4) was supplied to establish the +Pi and -Pi conditions, respectively. To detect the phosphatase activity, we added after sterilization ELF-97 reagent to a final concentration of 50 µM. Plants were grown in Percival chambers with continuous light, temperature 22 °C, and humidity around to 60% for 5 days. For phosphatase activity, we recorded gemmae development growth in high and low Pi conditions in the presence of ELF97. Confocal images were recorded at diverse time points by using CLSM Zeiss LM800 microscope, chlorophyll auto-florescence was observed at a 610 nm wavelength and ELF-97 was observed at 530 nm.

### 4.4. Free Phosphate Quantification

Free Pi was quantified by the malachite green assay [27], modified, and adapted measurements in young thalli. First, we ground the fresh samples (50 mg) in liquid nitrogen, added 500 µL of MES buffer (0.17 M, 100 mM DTT, pH 5.8) to each sample, and mixed vigorously. After centrifugation at 12,000 rpm for 10 min, the supernatant was collected in a new tube. Then, 50 µL of each sample were mixed with 115 μL of acid molybdate solution (17.55 g/L of ammonium molybdate tetrahydrate, 3 M sulfuric acid) and 700 μL of deionized water. Ten minutes later, 150 μL of green malachite solution (3.5 g/L polyvinyl alcohol, 0.35 g/L malachite green) were added. The absorbance was read at 610 nm after 2 h. A titration curve was performed in the same conditions, using a stock solution 1 mM KH2PO4 to measure 25, 12.5, 6.25, 3.13, 1.56, and 0 μM, respectively. Finally, to calculate the Pi concentration in each sample, we used a regression equation normalized with the respective data from fresh weight.

### 4.5. RNA Extraction, Sequencing, and Quantitative Real-Time qPCR Analysis

For RNA-seq, samples for RNA extraction (from two independent biological replicates per each condition and time point) were collected and ground in liquid nitrogen. For each sample, 900 μL of TRIzol reagent were added and incubated in ice for several minutes and 5 min at room temperature (RT). Previous to centrifugation at 13,000 rpm for 10 min at 4 °C, we add 200 μL of chloroform/isoamyl alcohol (24:1) and mixed briefly for 2 min at RT. The supernatant was collected in a new tube and incubated for 10 min at RT with 500 μL of isopropyl alcohol stored previously at −20 °C; later, it was precipitated by centrifugation at 13,000 rpm for 10 min at 4 °C. Finally, the precipitated RNA was washed with ethanol 75% (*v/v*), stored at −20 °C, and was resuspended in DEPC water. Before sequencing, the RNA samples were analyzed with Bioanalizer, and RIN values lower than 8 were discharged. Libraries were prepared using TruSeq RNA and sequenced with ILLUMINA next seq 1x75 for each replicate, time, and condition. For qPCR experiments, the total RNA was extracted and analyzed as described above. We used 5 μg of total RNA for each 20 μL reaction to obtain cDNA for each condition and time. cDNA was synthesized using SuperScript II (Invitrogen, Carlsbad, California, USA) according to the manufacturer’s protocol. The expression quantification was determined using SYBR Green PCR master mix (Applied Biosystems, MA, USA) in 10 μL reaction. The levels for each tested transcript were determined using three technical replicates per biological replicate and normalized to actin transcript levels. The results are represented as [2 ^(−∆ *C*t)^], the primers used in these reactions are listed in Figure S32.

### 4.6. Differential Expressed Genes Determination and GO Enrichment Analysis

The RNAseq dataset is available on the NCBI repository under the bioproject identifier PRJNA667150. We collected raw data to perform quality analysis with the software FastQC. In addition, the reads were processed with Trimmomatic software to remove the adapter contamination [94]. Then, we uploaded the filtered data to the Cyverse environment (https://de.cyverse.org/de/) and use the DNA subway tool (https://dnasubway.cyverse.org/) to generate RNAseq analysis to obtain the differentially expressed genes. We used the pipeline established for the tuxedo suit, the DEGs were selected based on FC (>1.5 and <−1.5) and FDR < 0.001. Venn diagrams were performed using the online tool (http://bioinformatics.psb.ugent.be/webtools/Venn/). On the other hand, the enrichment analysis of Gene Ontology (GO) terms was carried out for the DEG selected each time. The GO analysis was performed in the online tool *PlantRegMap* (http://plantregmap.gao-lab.org/go.php); we selected the *M. polymorpha* genome to perform the analysis of biological processes categories, and the *p*-value was lower than 0.001.

### 4.7. P1BS Search in DEGs

To identify the PHR1 DNA binding site (P1BS) in the cis-regulatory region of differentially expressed genes, we first retrieved a 3 kb fragment upstream of the start codon of all *M. polymorpha* genes detected in our transcriptomes using the v3.1 genome release available in Phytozome 13 (https://phytozome-next.jgi.doe.gov/). Then, we searched for the presence of P1BS in these cis-regulatory regions using HOMER (v4.11, 10-24-2019) [95]. For this, we used the P1BS consensus sequence (*GNATATNC*) to make a motif file with the seq2profile.pl tool, and then we queried the cis-regulatory regions of all the differentially expressed genes with the findMotifs.pl tool. The upstream regions of non-differentially expressed genes were selected as background for this analysis. Once we had the P1BS occurrences and their sequence, we built a sequence logo that represented the P1BS in *M. polymorpha* with ggseqlogo [96] in R.

### 4.8. Analysis of P1BS Enrichment

To determine if the P1BS was relatively enriched in the promoter set of differentially expressed genes compared with the promoter set of all the genes in our transcriptome, we used AME [68], a tool from the MEME suite [69]. We provided AME with the P1BS motif sequence to search and did two analyses in which we wanted to test for P1BS enrichment: the set of 3259 promoters with at least 1 P1BS obtained from the initial P1BS search with HOMER, and the 10,783 promoters belonging to non-differentially expressed genes. For both analyses, we utilized the 16,240 promoters available in the *Marchantia polymorpha* v3.1 genome release (https://phytozome.jgi.doe.gov) as a control sequence set. We selected an average odds score as a sequence scoring method and the Fisher’s exact test as the motif enrichment test. The list of promoters with P1BS enrichment is available on the Table S4.

## Supplementary Materials

The following are available online at https://www.mdpi.com/1422-0067/21/21/8354/s1, Figure S1: Species selected for the evolutionary analysis, Figures S2–S24: Phylogenetic reconstruction of genes related to Pi starvation response, Figure S25: Phenotypical response to Pi availability on marchantia polymorpha, Figure S26: Thallus promotes media acidification under low Pi availability, Figure S27: Transcriptional regulation of genes involved in organic acid 4 biosynthesis and exudation, Figure S28: Transcriptional regulation of genes involved in lipid turnover, Figure S29: Transcriptional regulation of peroxidases (MpPRXs) genes, Figure S30: Transcriptional regulation of NADPH oxidase (MpNOXs) genes, Figure S31: Transcriptional regulation of genes related to aronidin biosynthesis and Figure S32: Table for qPCR primers. Table S1: Gene ontology analysis of up-regulated genes at 12 HPT, Table S2: Gene ontology analysis of up-regulated genes at 24 HPT, Table S3: Gene ontology analysis of up-regulated genes at 150 HPT and Table S4: Enrichment analysis of P1BS motives.

## Abbreviations

Pi	Phosphate
P1BS	PHR1 binding sites
Ca	Calcium
Al	Aluminium
Fe	Iron
RSA	Root system architecture
OAs	Organic acids
TF	Transcription factor
IPP	Inositol polyphosphate
PM	Plasma membrane
+Pi	High phosphate availability
-Pi	Low phosphate availability
PSR	Phosphate starvation response
µM	Micromolar
HAS	Hours after sown
HPT	Hours post transference
DAS	Days after sown
FC	Fold change
DEGs	Differential expressed genes
FDR	False discovery rate
GO	Gene ontology
qPCR	Quantitative polymerase chain reaction
CREs	Cis regulatory elements
