# Supplementary material for: Transcriptional and Morpho-Physiological Responses of Marchantia polymorpha upon Phosphate Starvation
Source: Int J Mol Sci. 2020 Nov 7;21(21):8354. doi: 10.3390/ijms21218354 (PMC7672586; doi:10.3390/ijms21218354)
Supplement: Supplementary file 1 [file ijms-21-08354-s001.zip › Rico-Resendiz, etal 2020_Supplementary-information-fx.pdf]

Transcriptional and morpho-physiological responses of *Marchantia polymorpha* upon phosphate starvation

Felix Rico-Reséndiz[1], Sergio Alan Cervantes-Pérez[2], Annie Espinal-Centeno[1], Melissa Dipp-Álvarez[1], Araceli Oropeza-Aburto[2], Enrique Hurtado-Bautista[3], Andres Cruz-Hernández[4], John L. Bowman[5], Kimitsune Ishizaki[6], Mario A. Arteaga-Vázquez[7], Luis Herrera-Estrella[2,8] and Alfredo Cruz-Ramírez[1]*

Supplementary information:

phylogenetic reconstructions

To identify the putative orthologous genes involved in Pi starvation responses, we select a core of previous characterized genes from Arabidopsis as query. Their functions are related to Pi sensing, lipid turnover and transport. Furthemore, to cover the land phylogeny we select the 25 genomes distributed along viridiplantae (*Figure 1S*). Genome data was taken from phytozome (https://phytozome.jgi.doe.gov/pz/portal.html), *P. abies* genome project (http://congenie.org/blast) and fern base (https://www.fernbase.org/). After data collection, according to the procedure described in the methods section, we change the ID of each sequence using the first letter of the name in upper case followed by the first two letters of the second name in lowercase (eg. *Marchantia polymorpha* = Mpo). Then we assigned a continuous number to make a difference between protein members of the same family (ei. Mpo_001). Here, find you the phylogenetic reconstructions.

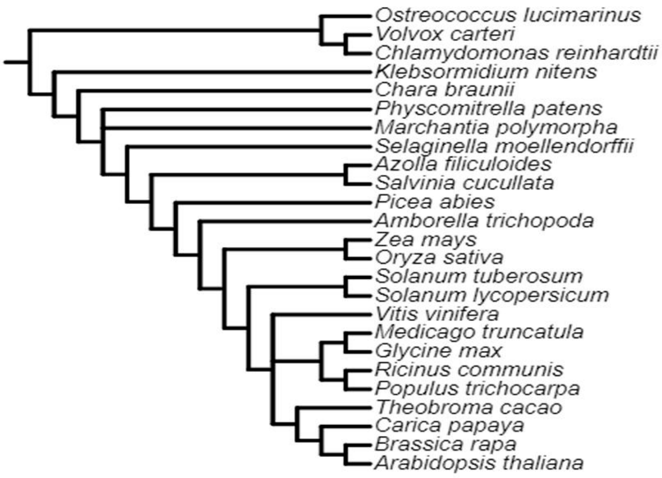

Figure 1S. Species selected for the evolutionary analysis

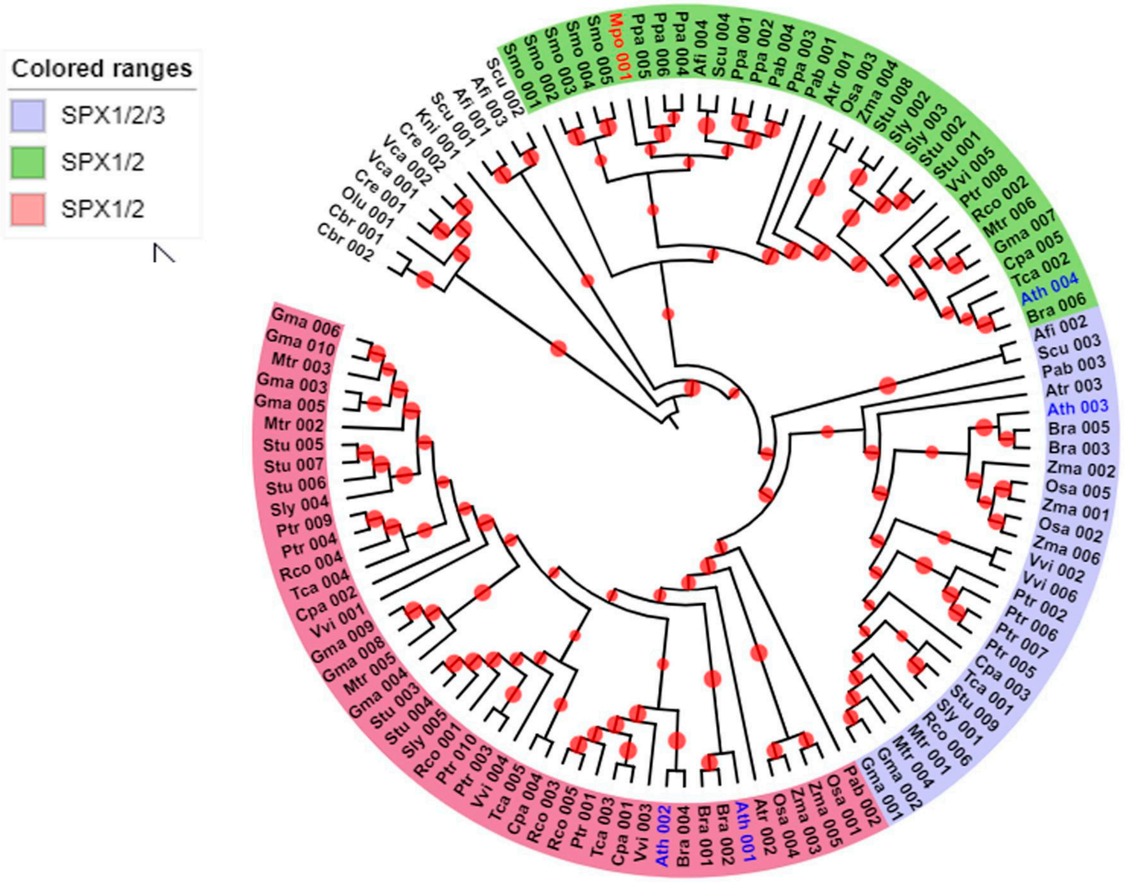

Figure 2S.- Phylogenetic reconstruction of the SPX class 1 family (ML-PROT-JTT 1000 bootstrap)**.**The SPX proteins of chlorophyte algae group in the base of tree as our outgroup. Close to Chlorophytes we found the sequences of streptophyte algae. In green we show the SPX4 clade, including the AtSPX4 from A. thaliana and MpSPX from M. polymorpha. then, the SPX3 clade is in blue and the SPX1/2 clade in pink. Labels of *A. thaliana* are colored in blue and *M. polymorpha* label is in red. The bootstrap support values have been shown in red circle, in a range from 60-100% which correlates with circle size.

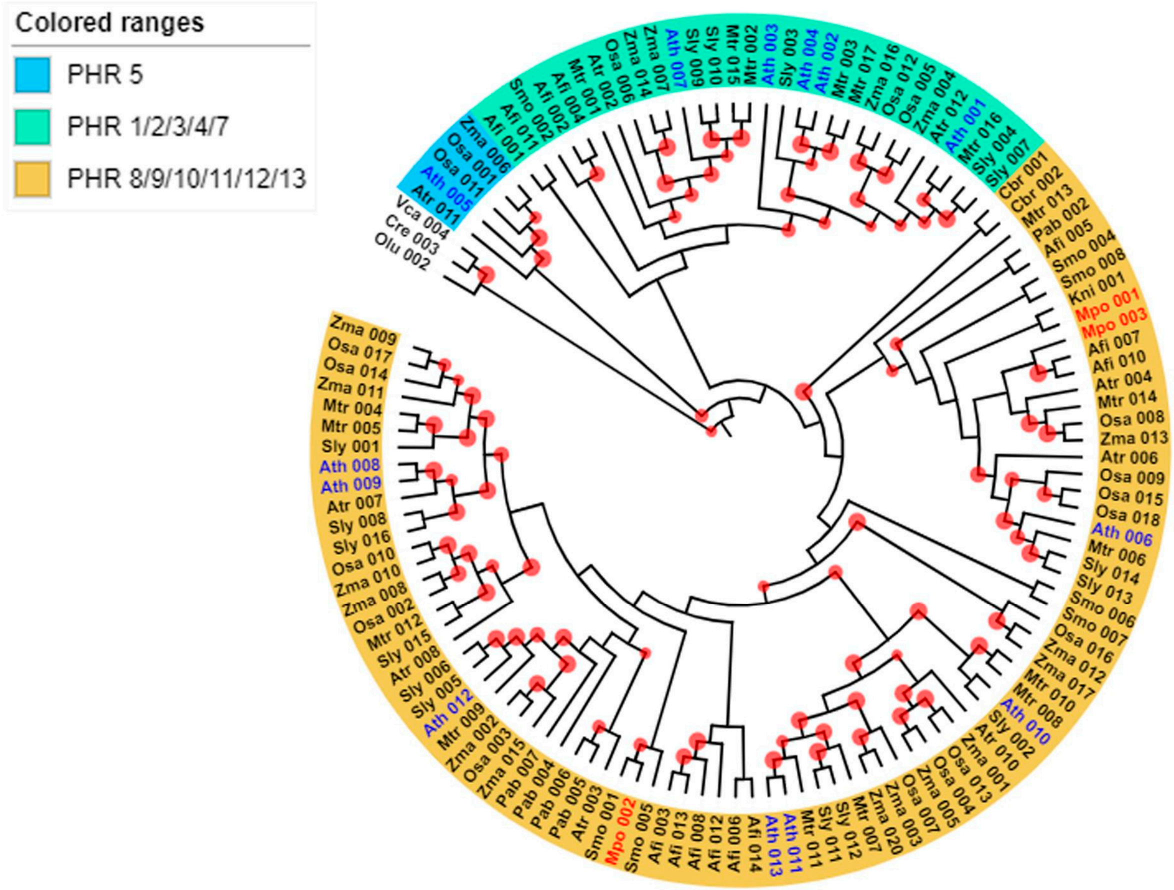

Figure 3S.- Phylogenetic reconstruction of the MYB-CC family (ML-PROT-JTT 1000 bootstrap)**.** The chlorophyte algae forms the outgroup at the base of the tree, here Cre_003 is the PSR1 gene. In blue light is the PHR5 clade, in green is the clade of PHR1/2/3/4/7 and in yellow the PHR 6/8/9/10/11/12/13. Labels of *A. thaliana* are colored in blue and *M. polymorpha* label is in red. The putative MpMYB-CC are in the yellow clade, MpPHR1 and MpPHR2 are closely related to AtPHR6 and MpPHR2 is close to AtPHR8/9/12. The bootstrap support values have been shown in red circle, in a range from 60-100% which correlates with circle size.

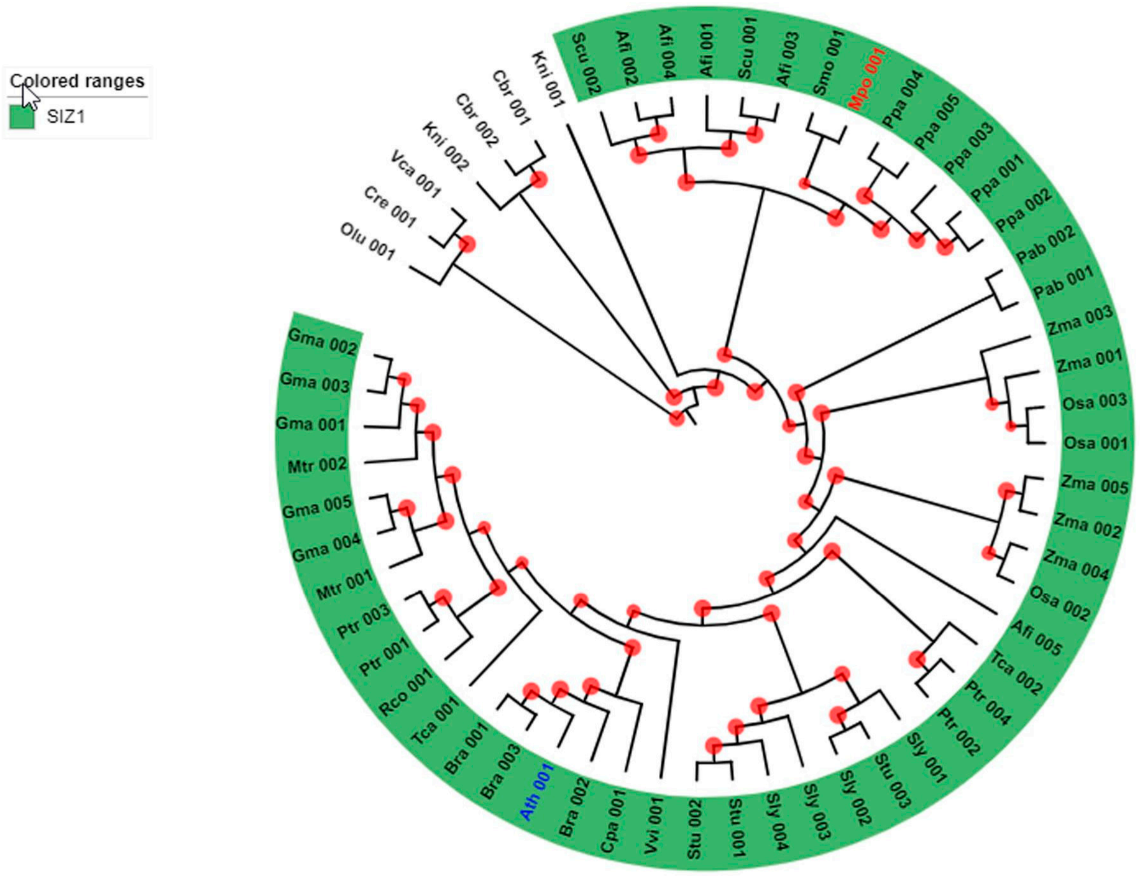

Figure 4S.- Phylogenetic reconstruction of the SIZ1 (PROT-JTT-I-F 1000 bootstrap replicates)**.**The phylogenetic reconstruction of SIZ1 correlates with the evolution of land plants. We find first the chlorophytes proteins, followed by the streptophyte algae sequences. The MpSIZ clusterize with sequences of *P. patens* and the early divergent vascular plants. Labels of *A. thaliana* are colored in blue and *M. polymorpha* label is in red. The bootstrap support values have been shown in red circle, in a range from 60-100% which correlates with circle size.

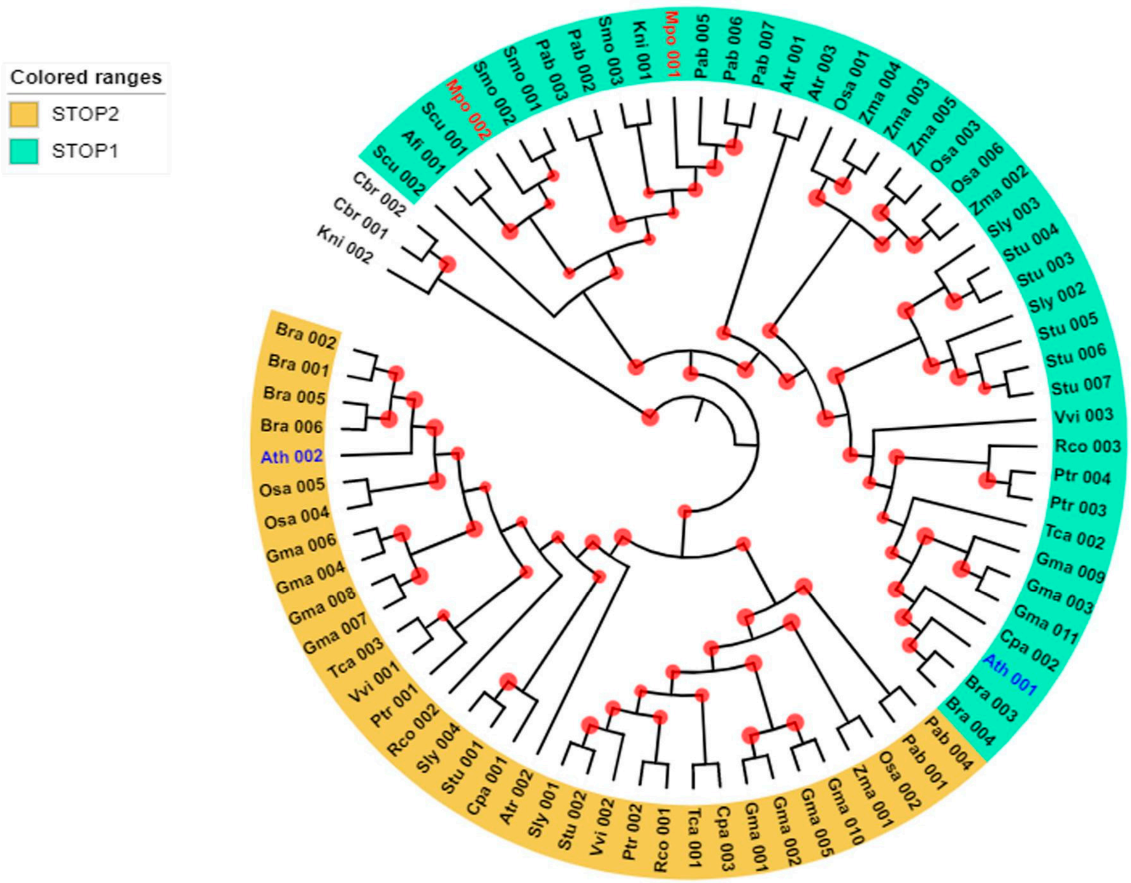

Figure 5S.- Phylogenetic reconstruction of the STOP1 (PROT-JTT-I-F 1000 bootstrap replicates)**.** In the chlorophyte genomes sampled we are not able to detect the putative orthologous proteins for STOP1. In this case, the out group is the streptophyte algae (label w/o color). Then phylogeny splits in two clades, green is STOP1 clade and yellow STOP2 clade. Into the STOP1 clade, we found the *M. polymorpha* sequences. The STOP2 clade only shows sequences since P. abie. Labels of *A. thaliana* are colored in blue and *M. polymorpha* label is in red. The bootstrap support values have been shown in red circle, in a range from 60-100% which correlates with circle size.

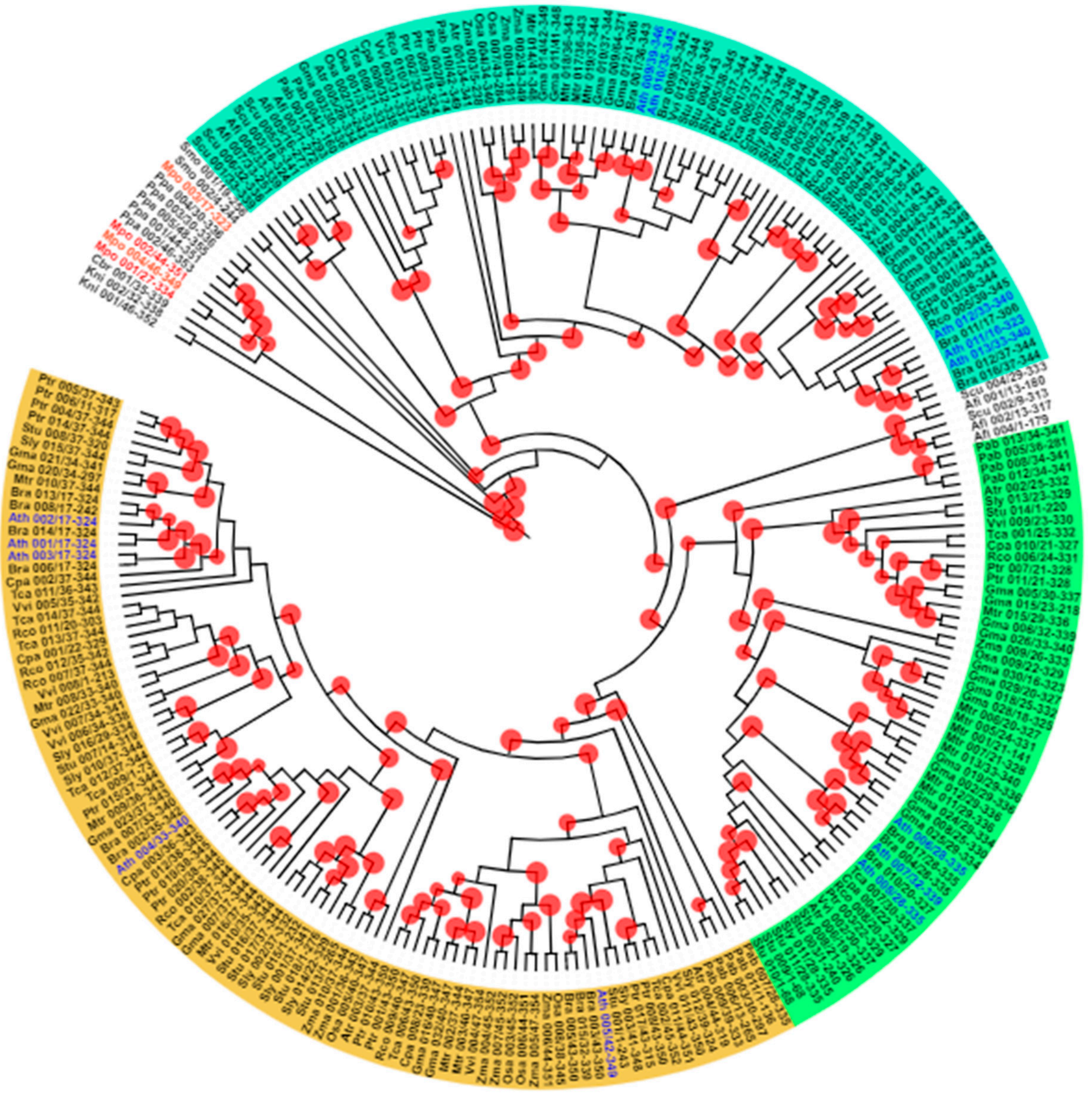

Figure 6S.- Phylogenetic reconstruction of the ALMT1 (PROT-JTT-I-F 1000 bootstrap replicates)**.** The sequences from non vascular plants and *S. moellendorffii* form an out group, when we found the four locus of *M. polymorpha*. then we found three major clades, in blue clade we found the *AtALMT9/10/11/12/13*. The green clade contains the *AtALMT6/7/8* and in yellow *AtALMT1/2/3/4/5*. Labels of *A. thaliana* are colored in blue and *M. polymorpha* label is in red. The bootstrap support values have been shown in red circle, in a range from 60-100% which correlates with circle size.

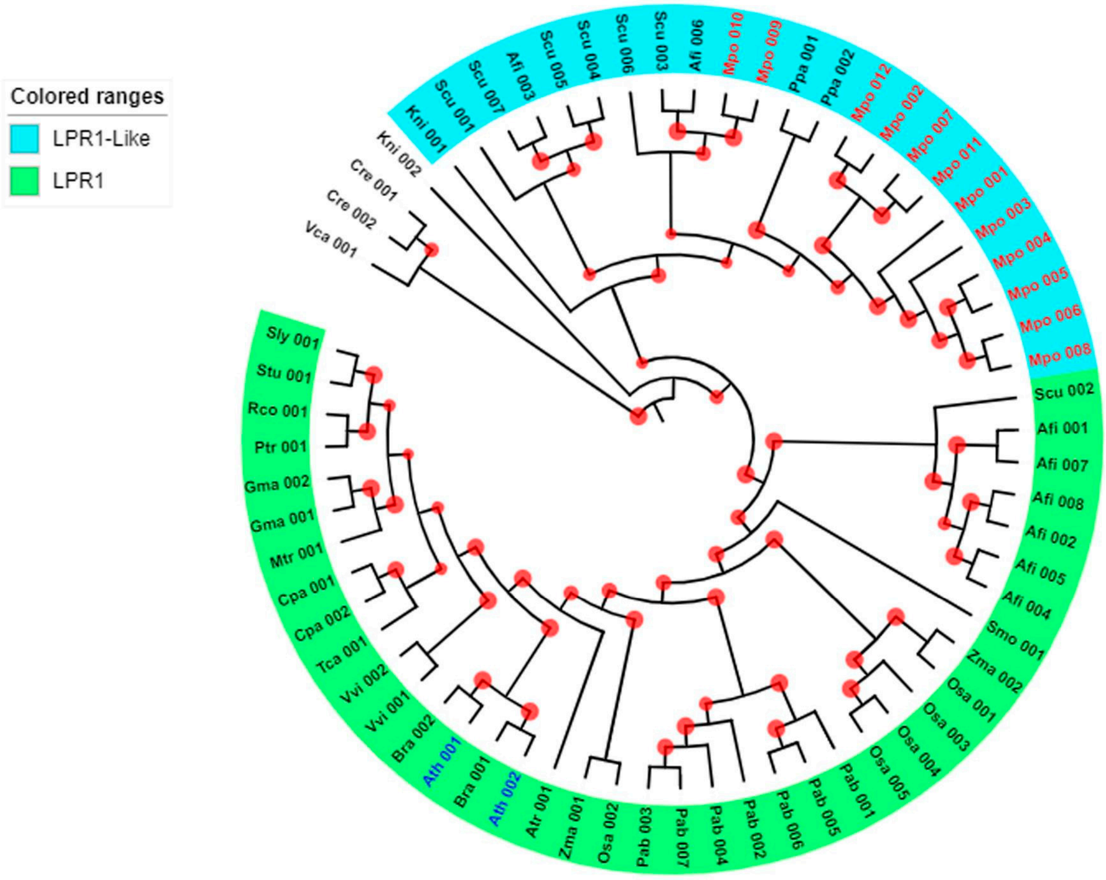

Figure 7S.- Phylogenetic reconstruction of LPR-L (PROT-JTT-I-F 1000 bootstrap replicates)**.**The phylogenetic reconstruction of LPR1 show two clades, the blue clade named LPR1-Like contain sequences from bryophytes to ferns and the other clade contain sequences since ferns to angiosperms. Labels of *A. thaliana* are colored in blue and *M. polymorpha* label is in red. The bootstrap support values have been shown in red circle, in a range from 60-100% which correlates with circle size.

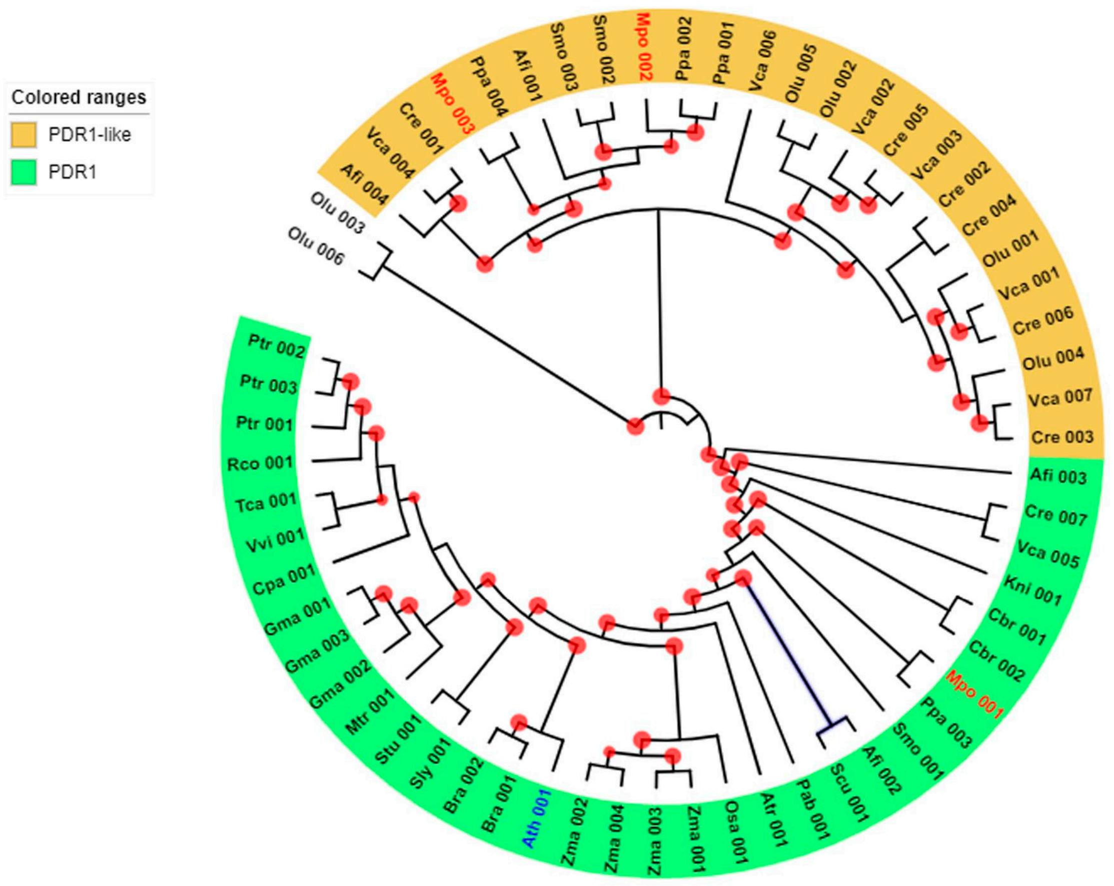

Figure 8S.- Phylogenetic reconstruction of the PDR2 (PROT-JTT-I-F 1000 bootstrap replicates)**.** The phylogenetic reconstruction clusterize the sequences in two clades, named PDR2-Like (Yellow) and PDR2 (Green). The PDR2-Like clade contains sequences from green algae to ferns, but not for flowering plants. The PDR2 clade displays sequences from green algae to flowering plants. Labels of *A. thaliana* are colored in blue and *M. polymorpha* label is in red. The bootstrap support values have been shown in red circle, in a range from 60-100% which correlates with circle size.

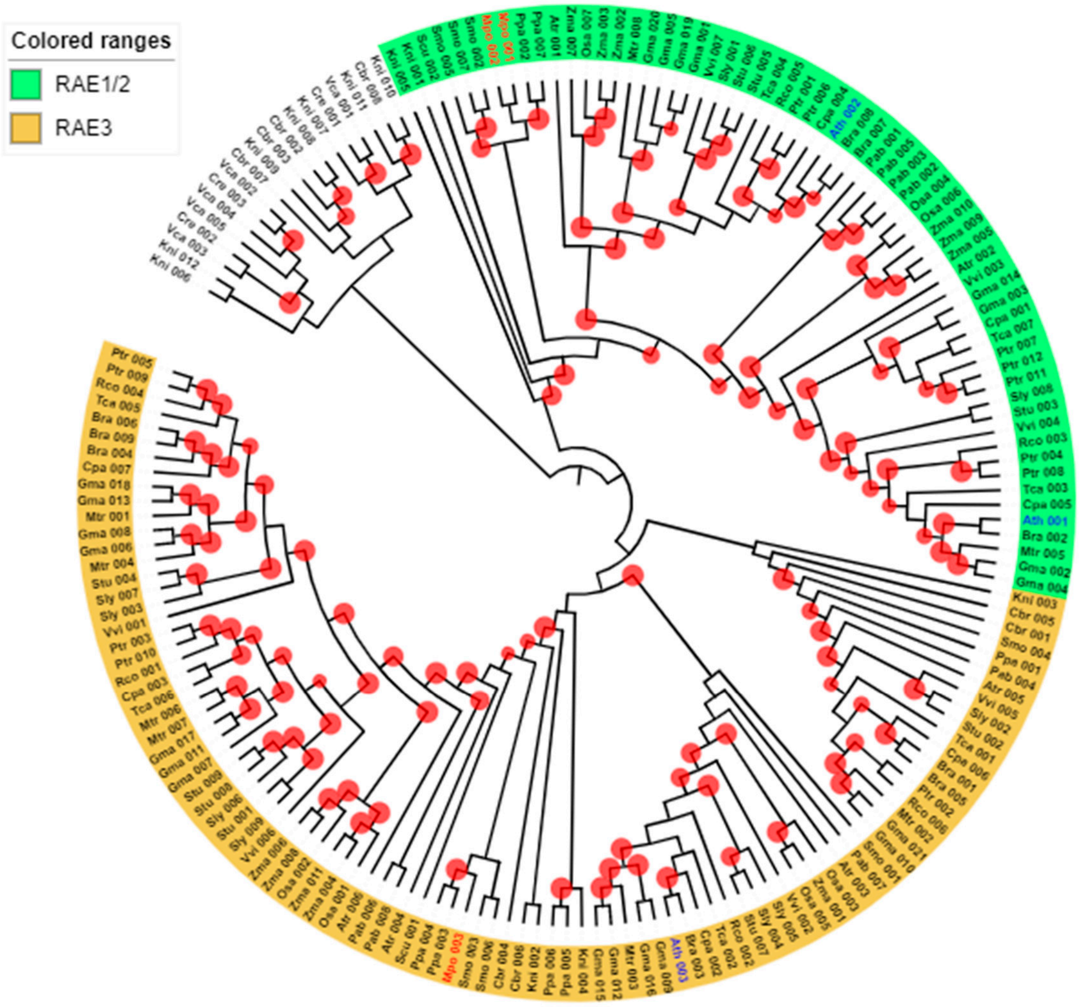

Figure 9S.- Phylogenetic reconstruction of the RAE (PROT-JTT-I-F 1000 bootstrap replicates)**.** The phylogenetic reconstruction clusterize the sequences in two clades, named RAE1/2 (Green) and RAE3 (Yellow). The *M. polymorpha* sequences *MpRAE1/2* belong to the clade of RAe1/2 and are the most putative orthologous genes to *AtRAE1*. While the *MpRAE3* is clusterized with the *AtRAE3*. Labels of *A. thaliana* are colored in blue and *M. polymorpha* label is in red. The bootstrap support values have been shown in red circle, in a range from 60-100% which correlates with circle size.

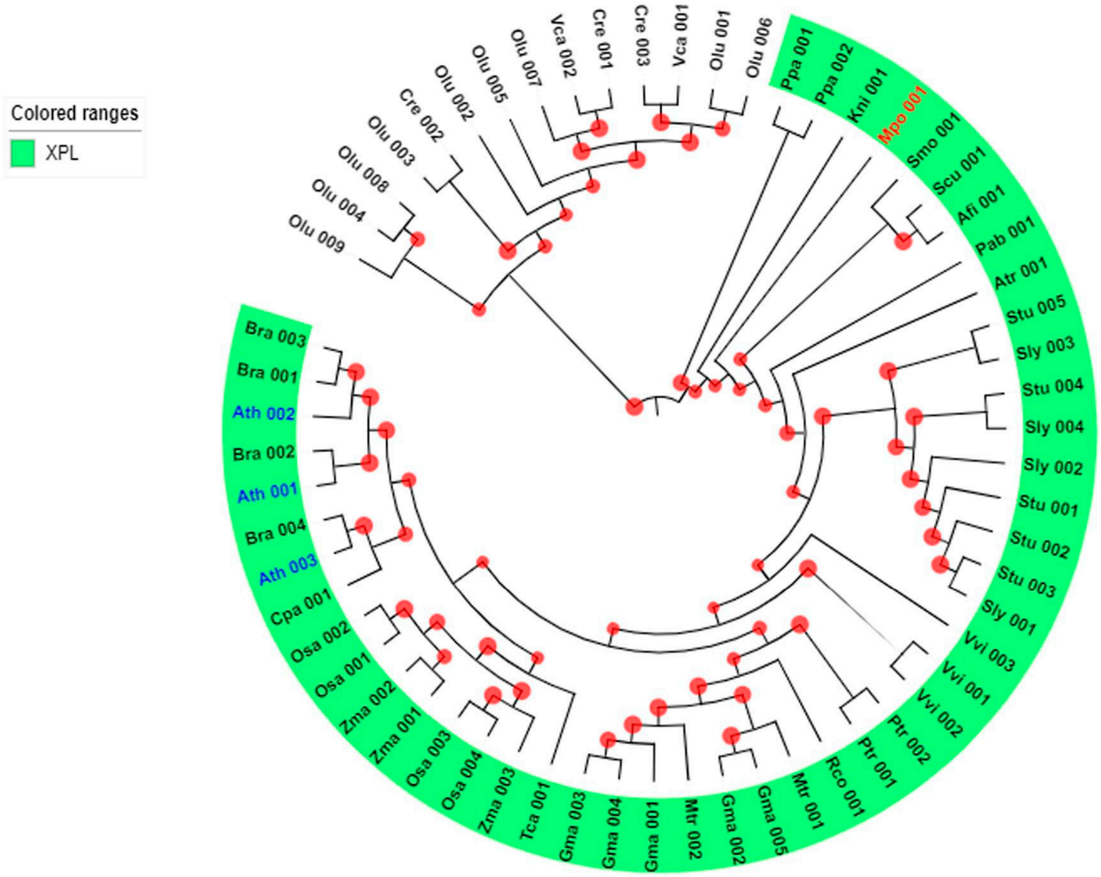

Figure 10S.- Phylogenetic reconstruction of the XPL (PROT-JTT-I-F 1000 bootstrap replicates)**.** The phylogenetic reconstruction shows two clades, one for the chlorophyte genomes and other in green for the streptophytes. Labels of *A. thaliana* are colored in blue and *M. polymorpha* label is in red. The bootstrap support values have been shown in red circle, in a range from 60-100% which correlates with circle size.

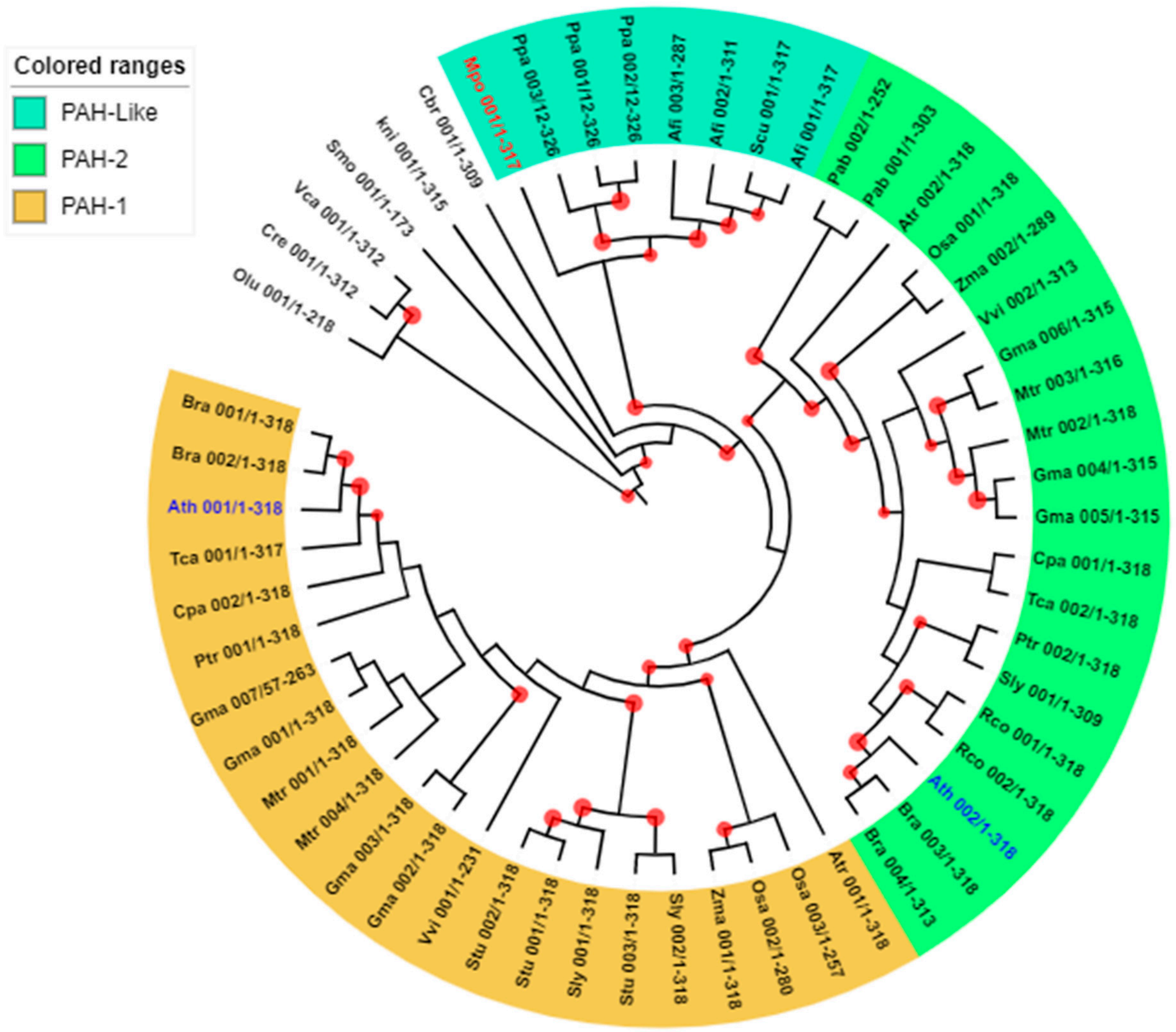

Figure 11S.- Phylogenetic reconstruction of the PAH (PROT-JTT-I-F 1000 bootstrap replicates)**.** The phylogenetic reconstruction shows two sister clades for the *AtPAH1* and *AtPAH2* in yellow and green ,respectively. the *MpPAH* forms another clade with sequences from bryophytes, early divergent vascular plants, in blue. Labels of *A. thaliana* are colored in blue and *M. polymorpha* label is in red. The bootstrap support values have been shown in red circle, in a range from 60-100% which correlates with circle size.

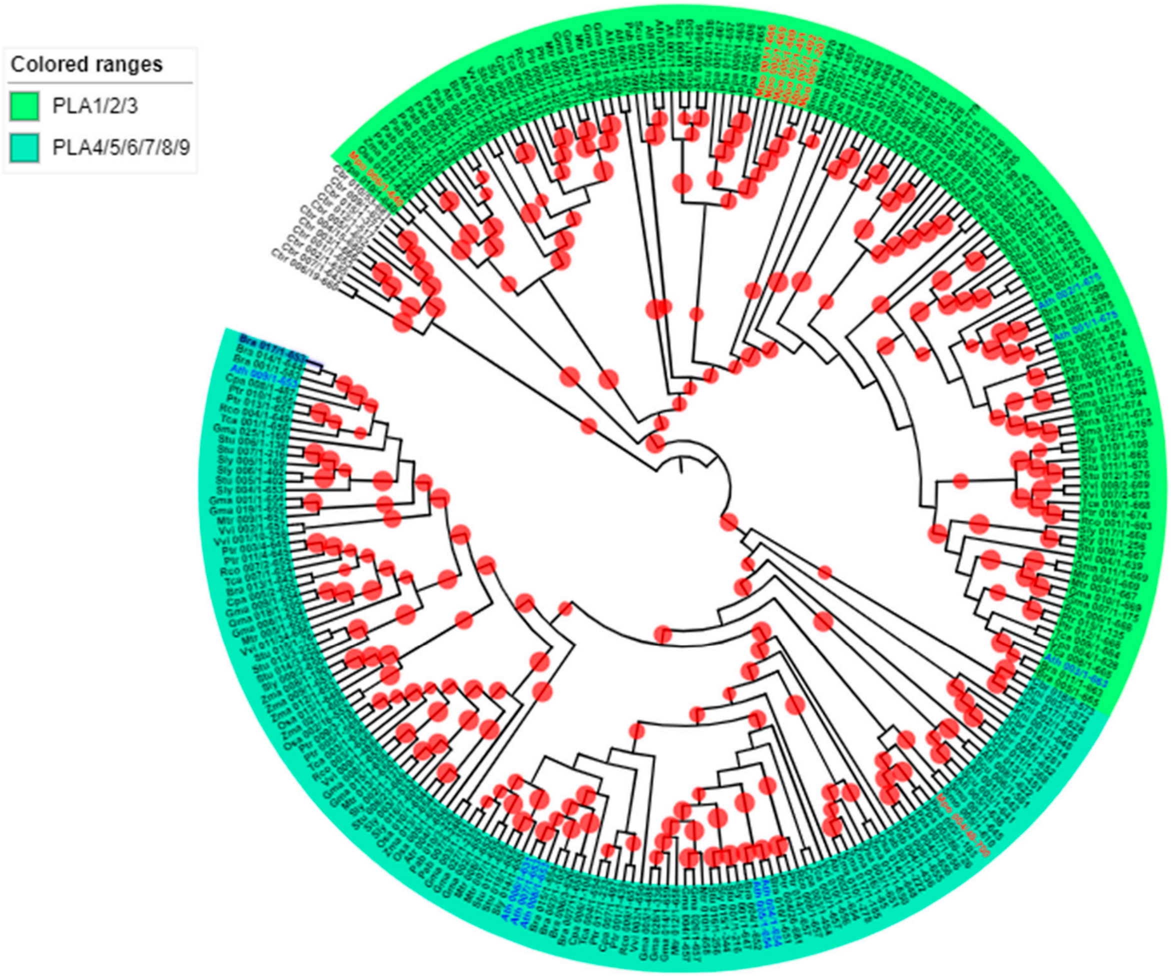

Figure 12S.- Phylogenetic reconstruction of the PLA (PROT-JTT-I-F 1000 bootstrap replicates)**.** The phylogenetic reconstruction shows two sister clades for the *AtPLA1/2/3* and *AtPA4/5/6/7/8/9* in green and blue ,respectively. Labels of *A. thaliana* are colored in blue and *M. polymorpha* label is in red. The bootstrap support values have been shown in red circle, in a range from 60-100% which correlates with circle size.

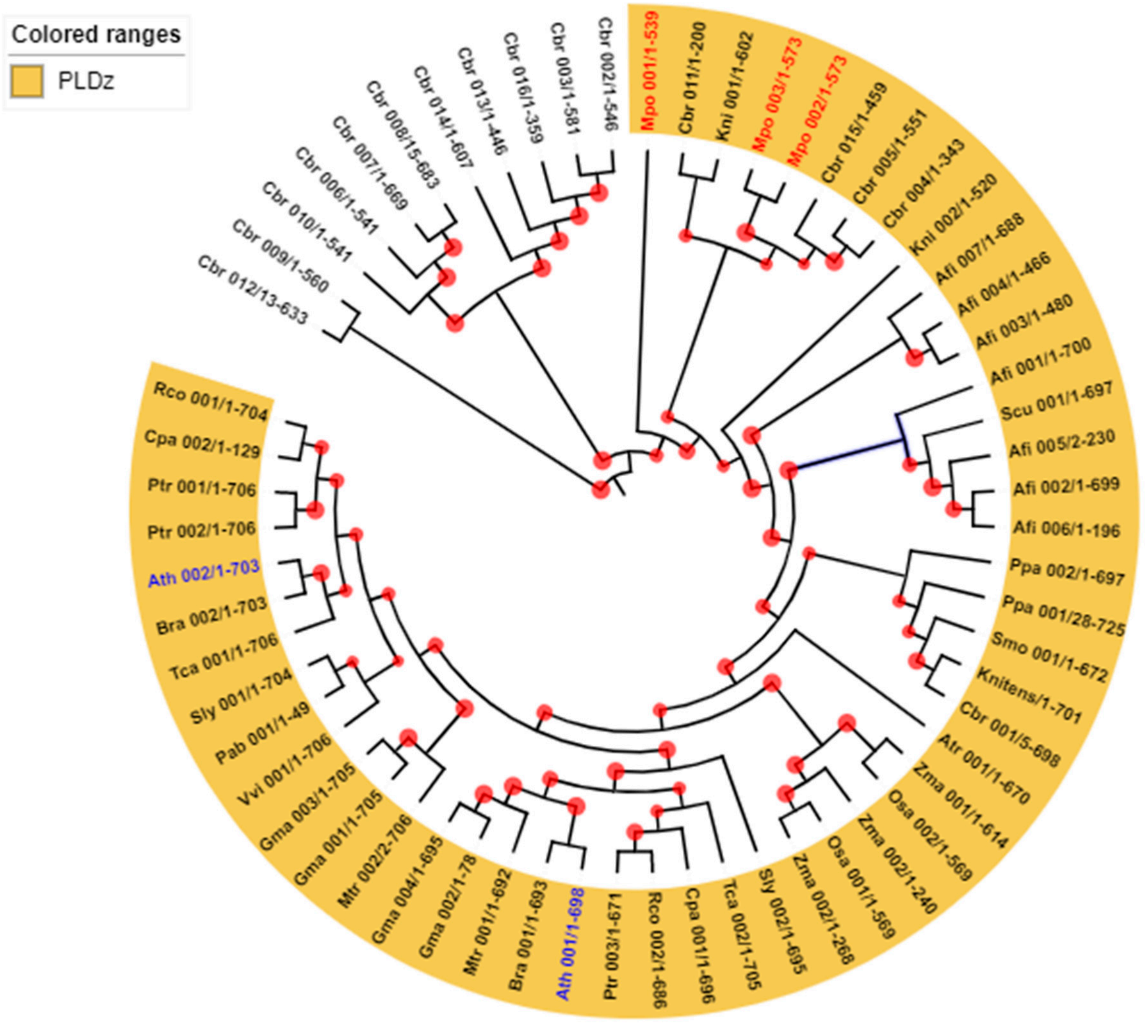

Figure 13S.- Phylogenetic reconstruction of the PLDz (PROT-JTT-I-F 1000 bootstrap replicates)**.** The phylogenetic reconstruction shows two sister clades for the *AtPLDs1/2/3* and *AtPA4/5/6/7/8/9* in green and blue ,respectively. Labels of *A. thaliana* are colored in blue and *M. polymorpha* label is in red. The bootstrap support values have been shown in red circle, in a range from 60-100% which correlates with circle size.

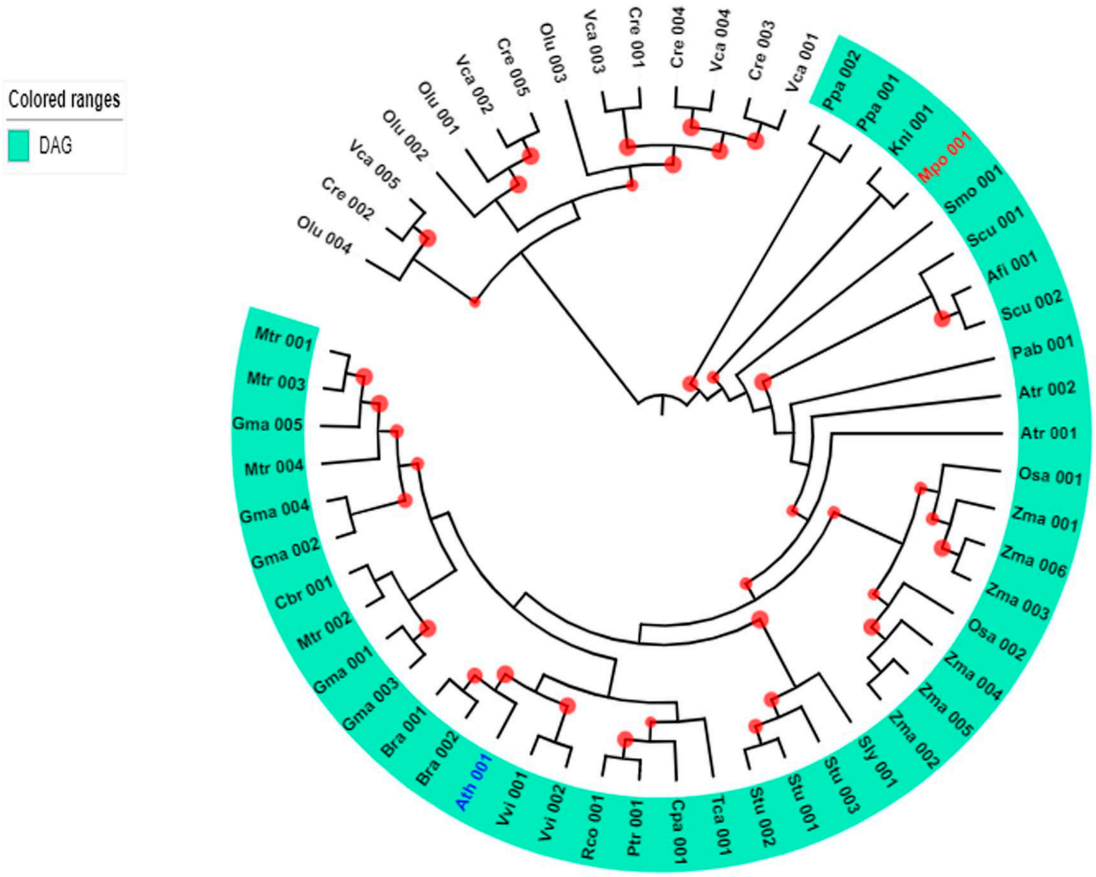

Figure 14S.- Phylogenetic reconstruction of the DAG (PROT-JTT-I-F 1000 bootstrap replicates)**.** The phylogenetic reconstruction for DAG shows two clades, chlorophytes and streptophytes (Blue) respectively. Labels of *A. thaliana* are colored in blue and *M. polymorpha* label is in red. The bootstrap support values have been shown in red circle, in a range from 60-100% which correlates with circle size.

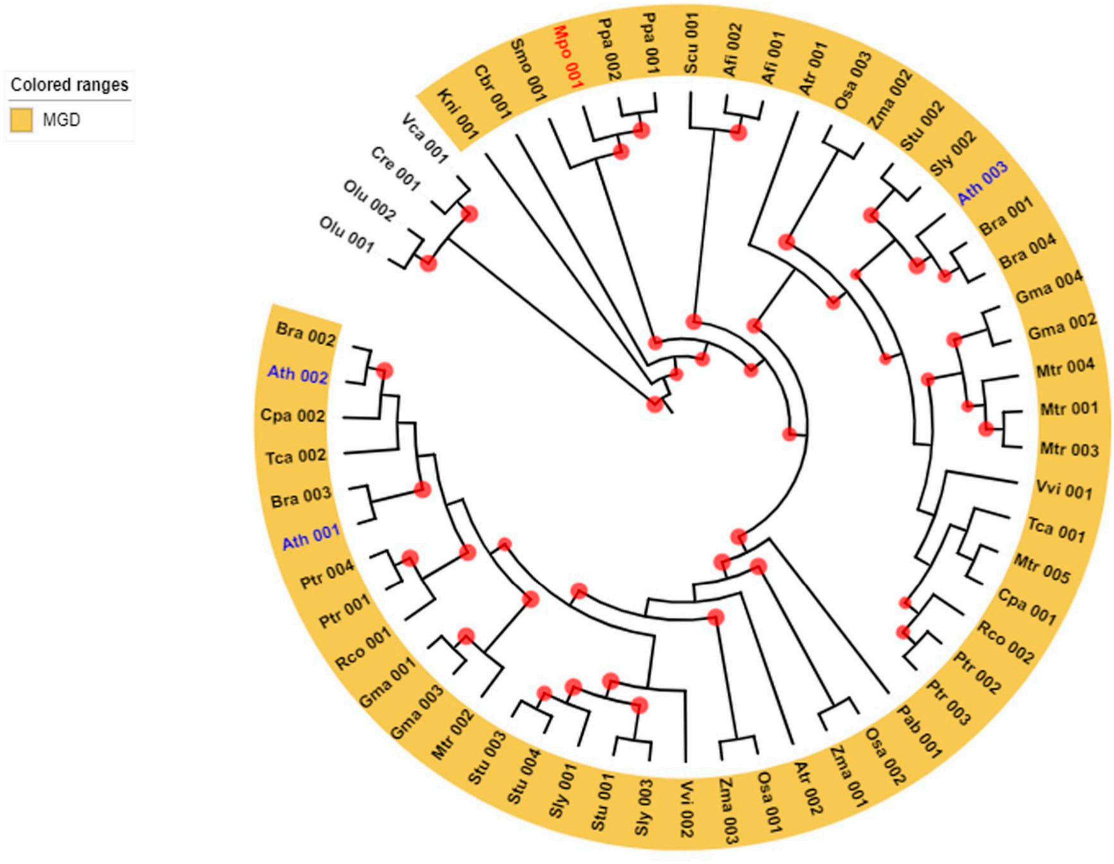

Figure 15S.- Phylogenetic reconstruction of the MGD (PROT-JTT-I-F 1000 bootstrap replicates)**.** The phylogenetic reconstruction clusterize in two clades, one includes sequences from chlorophyte genomes. The other contains the sequences of streptophytes, into the clade, we found two subclades for the flowering plant sequences. Labels of *A. thaliana* are colored in blue and *M. polymorpha* label is in red. The bootstrap support values have been shown in red circle, in a range from 60-100% which correlates with circle size.

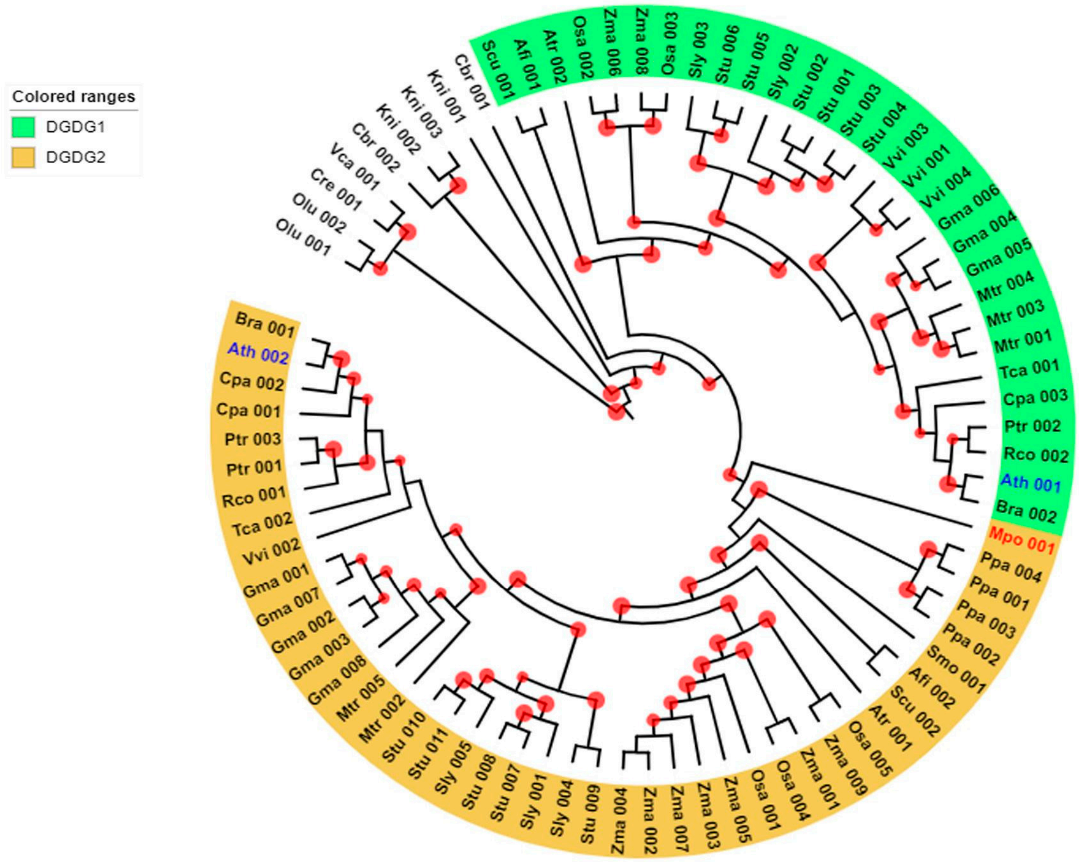

Figure 16S.- Phylogenetic reconstruction of the DGDG (PROT-JTT-I-F 1000 bootstrap replicates)**.** The phylogenetic reconstruction shows that embryophyte sequences form two clades, DGDG1 in green contain sequences exclusively of vascular plants. Whereas the DGDG2 in yellow contains sequences of bryophytes. Labels of *A. thaliana* are colored in blue and *M. polymorpha* label is in red. The bootstrap support values have been shown in red circle, in a range from 60-100% which correlates with circle size.

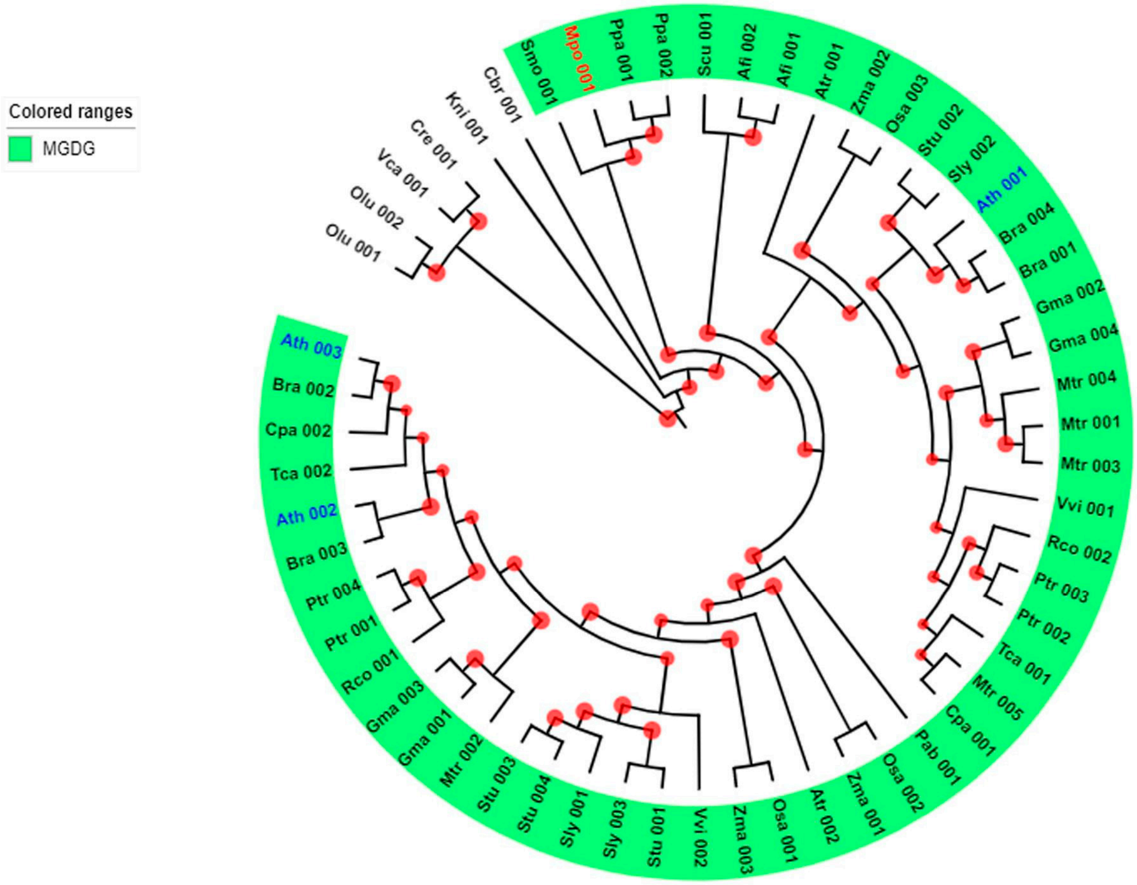

Figure 17S.- Phylogenetic reconstruction of the MGDG (PROT-JTT-I-F 1000 bootstrap replicates)**.** The phylogenetic reconstruction clusterize the MGDG sequences of embryophytes in a single clade (green), however the flowering plants form two subclades. Labels of *A. thaliana* are colored in blue and *M. polymorpha* label is in red. The bootstrap support values have been shown in red circle, in a range from 60-100% which correlates with circle size.

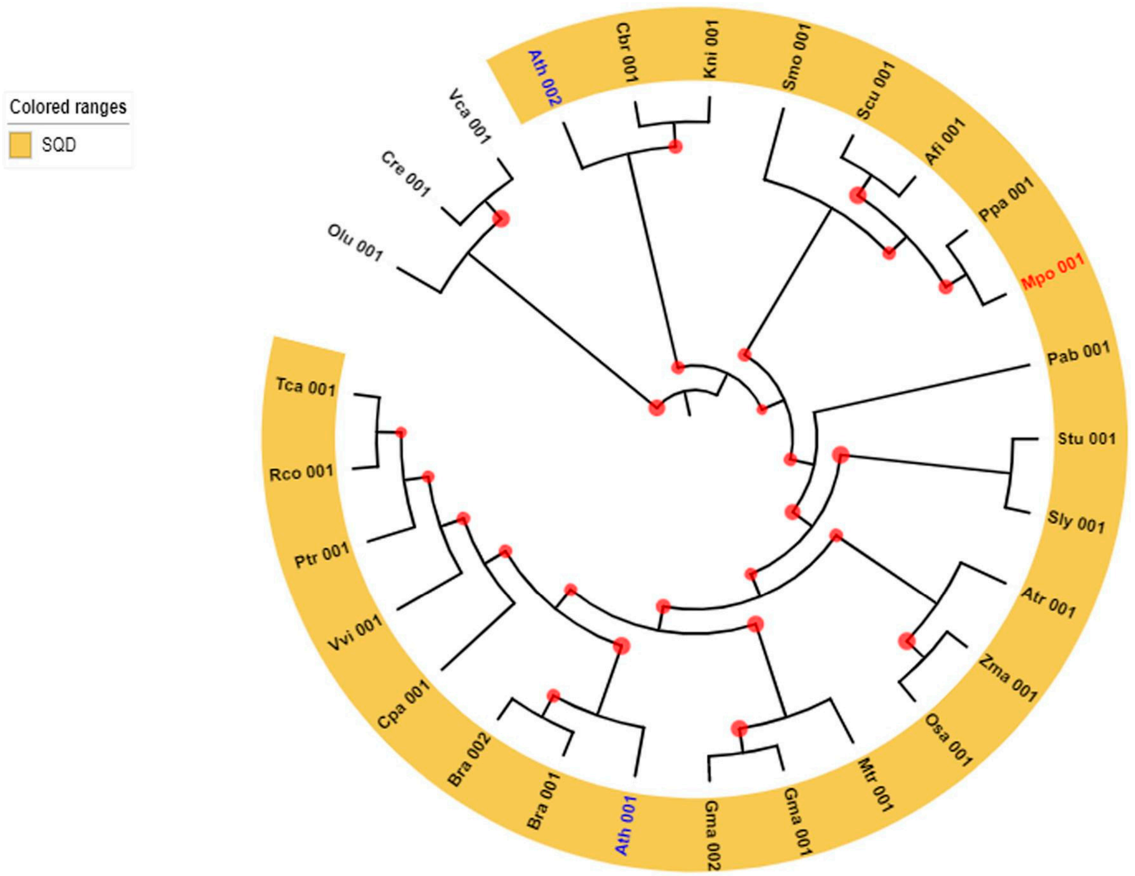

Figure 18S.- Phylogenetic reconstruction of the SQD (PROT-JTT-I-F 1000 bootstrap replicates)**.** The phylogenetic reconstruction shows a single clade, for SQD sequences which clusterize according to the species tree. Labels of *A. thaliana* are colored in blue and *M. polymorpha* label is in red. The bootstrap support values have been shown in red circle, in a range from 60-100% which correlates with circle size.

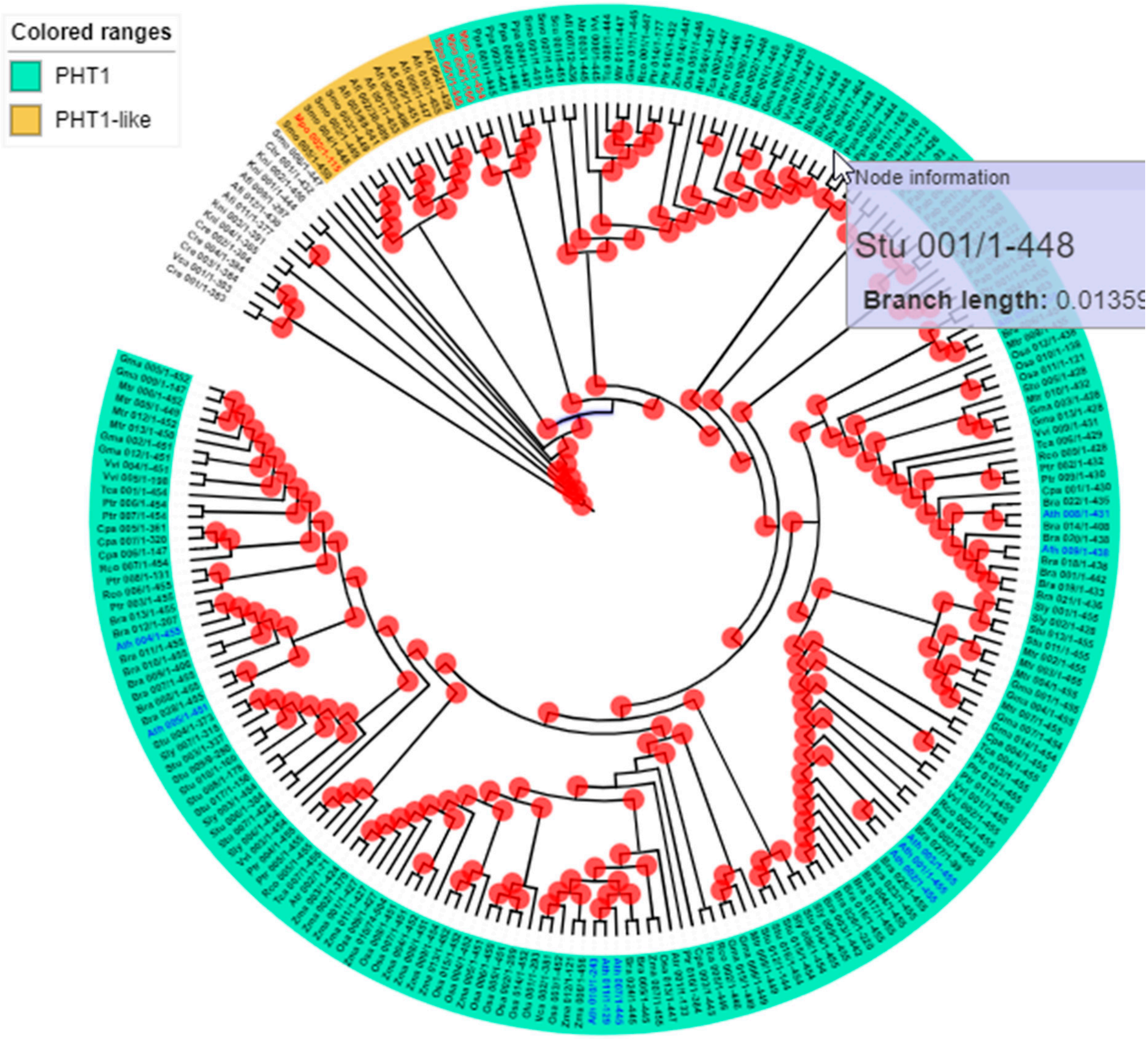

Figure 19S.- Phylogenetic reconstruction of the PHT (PROT-JTT-I-F 1000 bootstrap replicates)**.** The phylogenetic reconstruction shows two clades, in blue the PHT1 and in yellow the PHT1-like. *MpPHT1/3/4* belongs to PHT1 clades and *MpPHT2* to PHT1-Like. Labels of *A. thaliana* are colored in blue and *M. polymorpha* label is in red. The bootstrap support values have been shown in red circle, in a range from 60-100% which correlates with circle size.

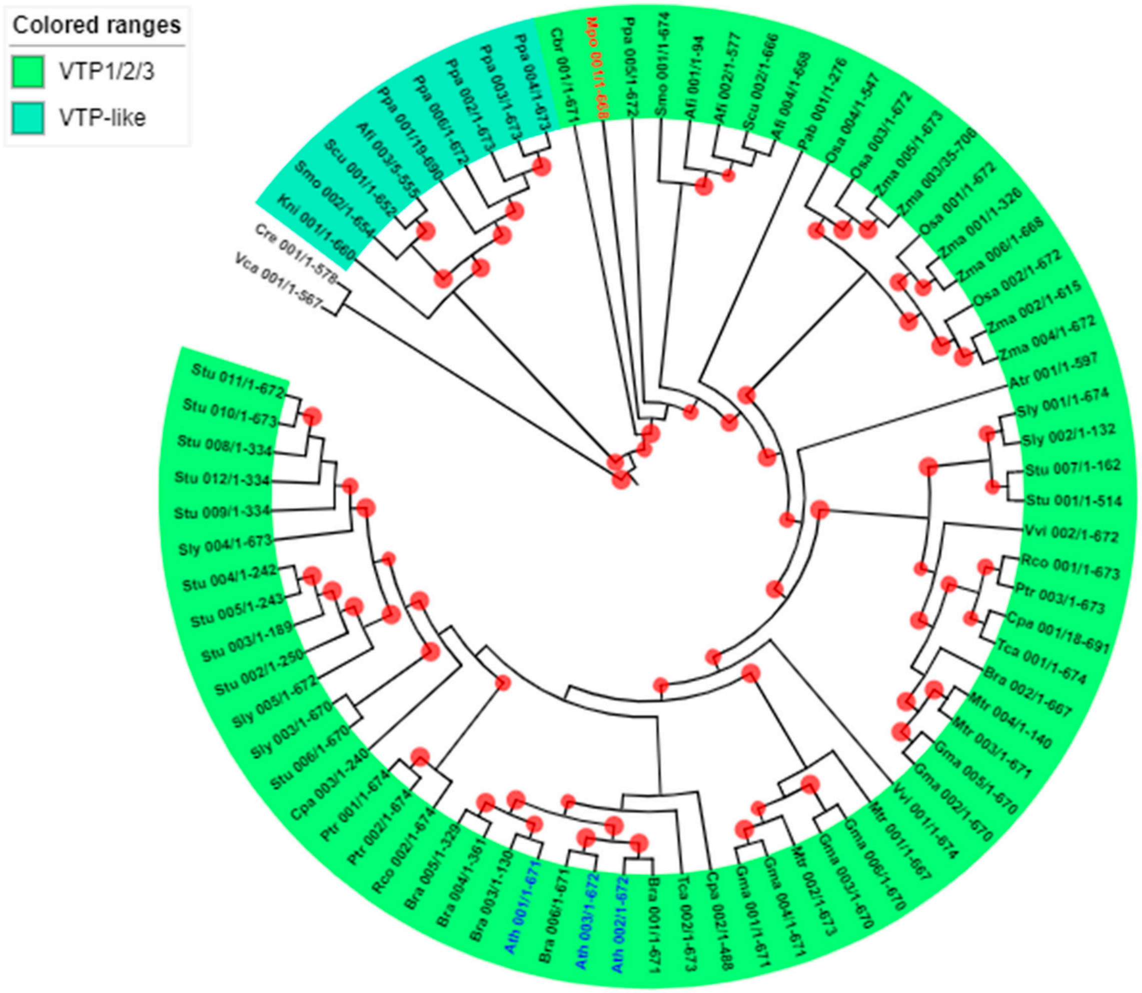

Figure 20S.- Phylogenetic reconstruction of the VTP1 (PROT-JTT-I-F 1000 bootstrap replicates)**.** The phylogenetic reconstruction shows two clades, in blue the VTP-like and in green the VTP1/2/3. Labels of *A. thaliana* are colored in blue and *M. polymorpha* label is in red. The bootstrap support values have been shown in red circle, in a range from 60-100% which correlates with circle size.

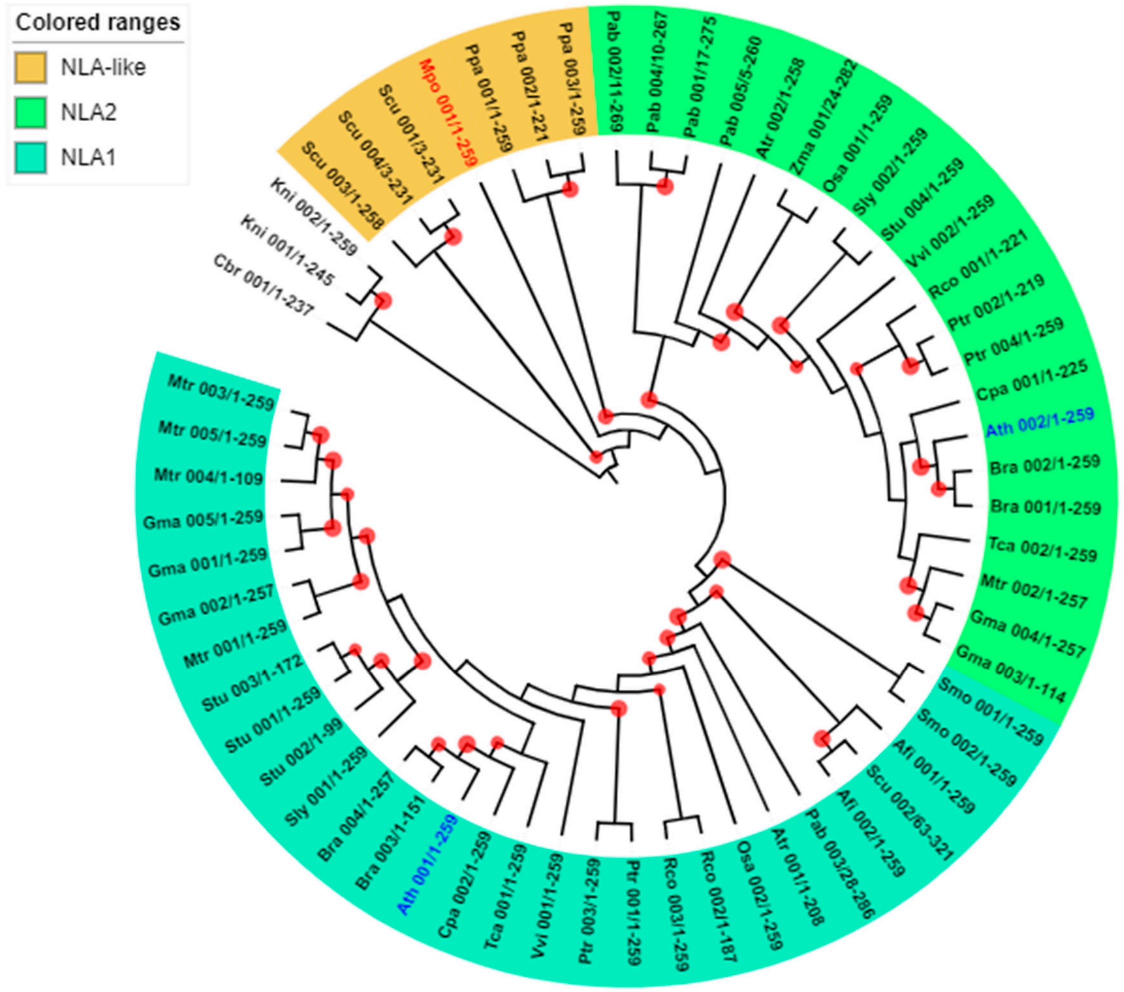

Figure 21S.- Phylogenetic reconstruction of the NLA (PROT-JTT-I-F 1000 bootstrap replicates)**.** The phylogenie shows two clades for AtNLA1 and AtNLA2 in blue and green respectively. Also, additional clades named NLA-like (Yellow) contain sequences from bryophytes. Labels of *A. thaliana* are colored in blue and *M. polymorpha* label is in red. The bootstrap support values have been shown in red circle, in a range from 60-100% which correlates with circle size.

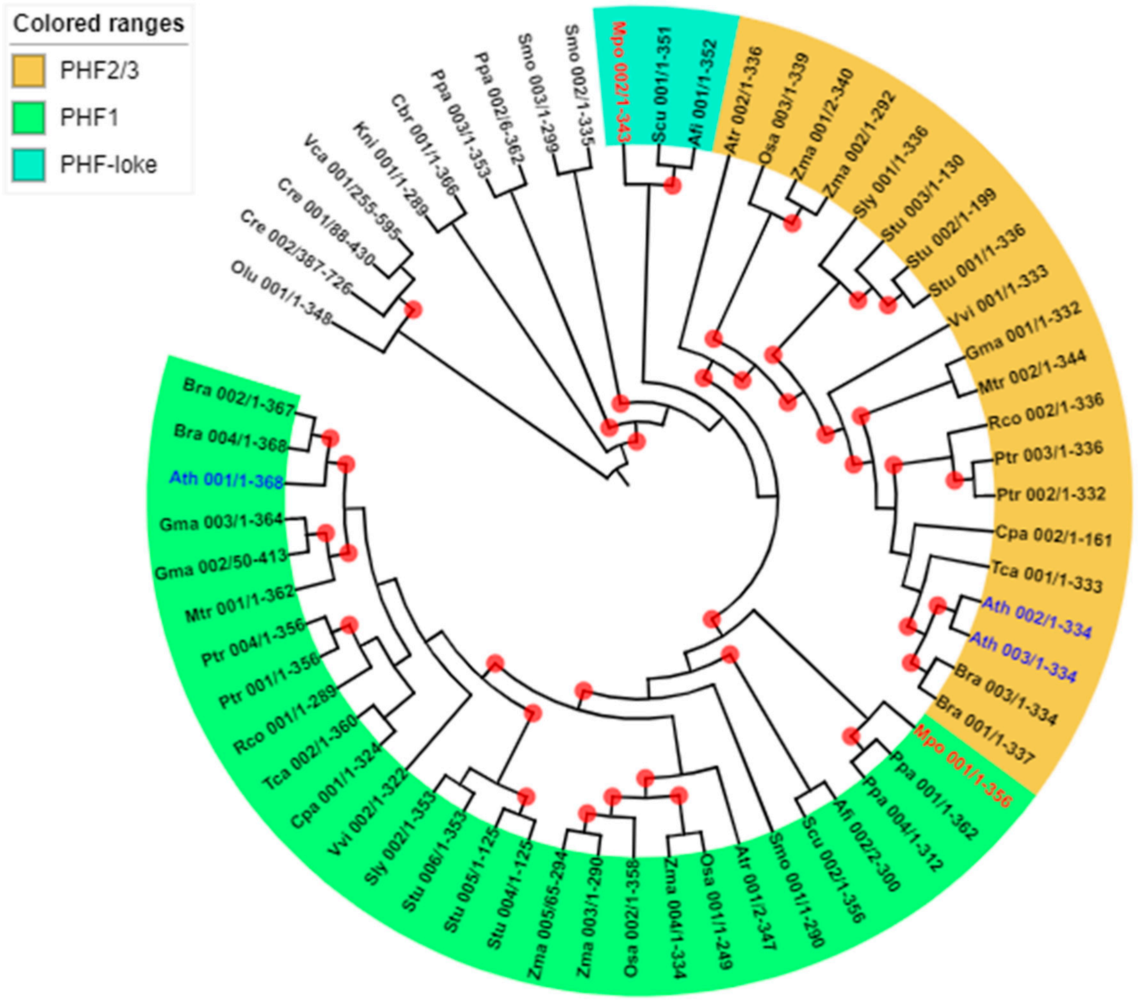

Figure 22S.- Phylogenetic reconstruction of the PHF (PROT-JTT-I-F 1000 bootstrap replicates)**.** The phylogeny shows two clades for *AtPHF1* and *AtPHF2/3* in green and yellow respectively. Also, additional clades named *PHF-like* (Blue) contain sequences from bryophytes. *MpPHF1-L* is the most putative orthologous gene of *AtPHF1*. Labels of *A. thaliana* are colored in blue and *M. polymorpha* label is in red. The bootstrap support values have been shown in red circle, in a range from 60-100% which correlates with circle size.

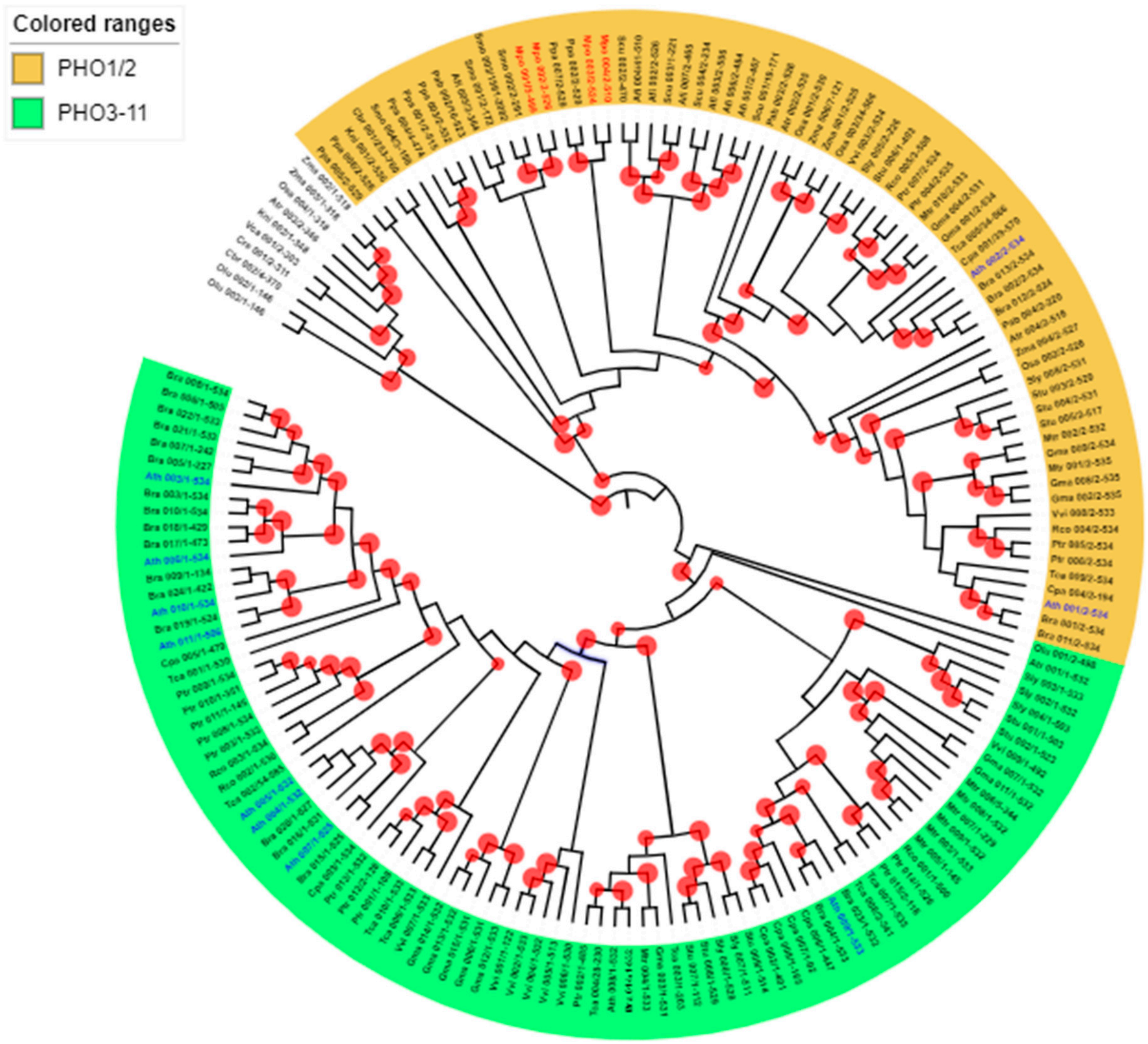

Figure 23S.- Phylogenetic reconstruction of the PHO1 (PROT-JTT-I-F 1000 bootstrap replicates)**.** The phylogenetic reconstruction clusterize two clades for *AtPHO1/3* and *AtPHO3-11* in yellow and green, respectively. Labels of *A. thaliana* are colored in blue and *M. polymorpha* label is in red. The bootstrap support values have been shown in red circle, in a range from 60-100% which correlates with circle size.

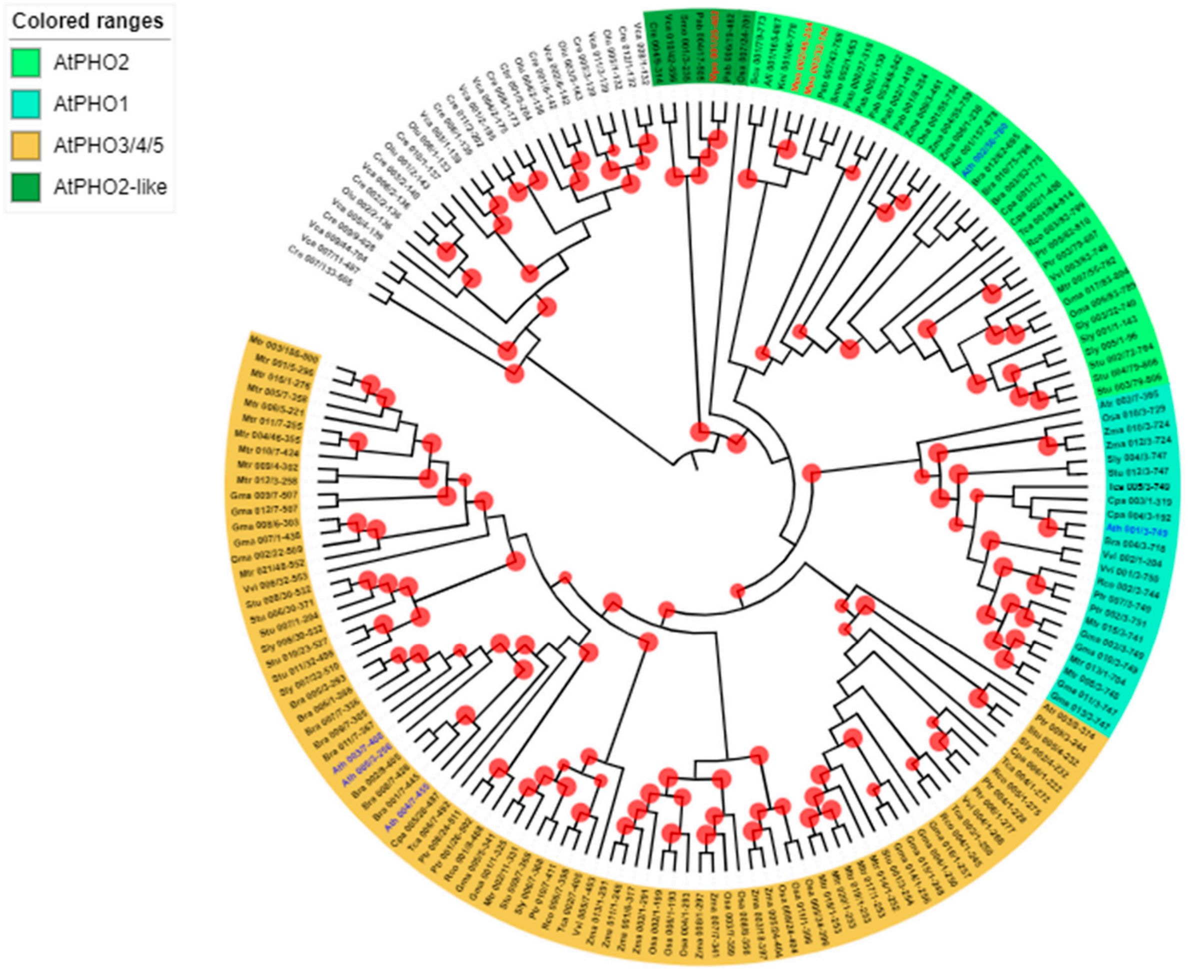

Figure 24S.- Phylogenetic reconstruction of the PHO2 (PROT-JTT-I-F 1000 bootstrap replicates)**.** The phylogenetic reconstruction clusterize two clades for *AtPHO1/3* and *AtPHO3-11* in yellow and green, respectively. Labels of *A. thaliana* are colored in blue and *M. polymorpha* label is in red. The bootstrap support values have been shown in red circle, in a range from 60-100% which correlates with circle size.

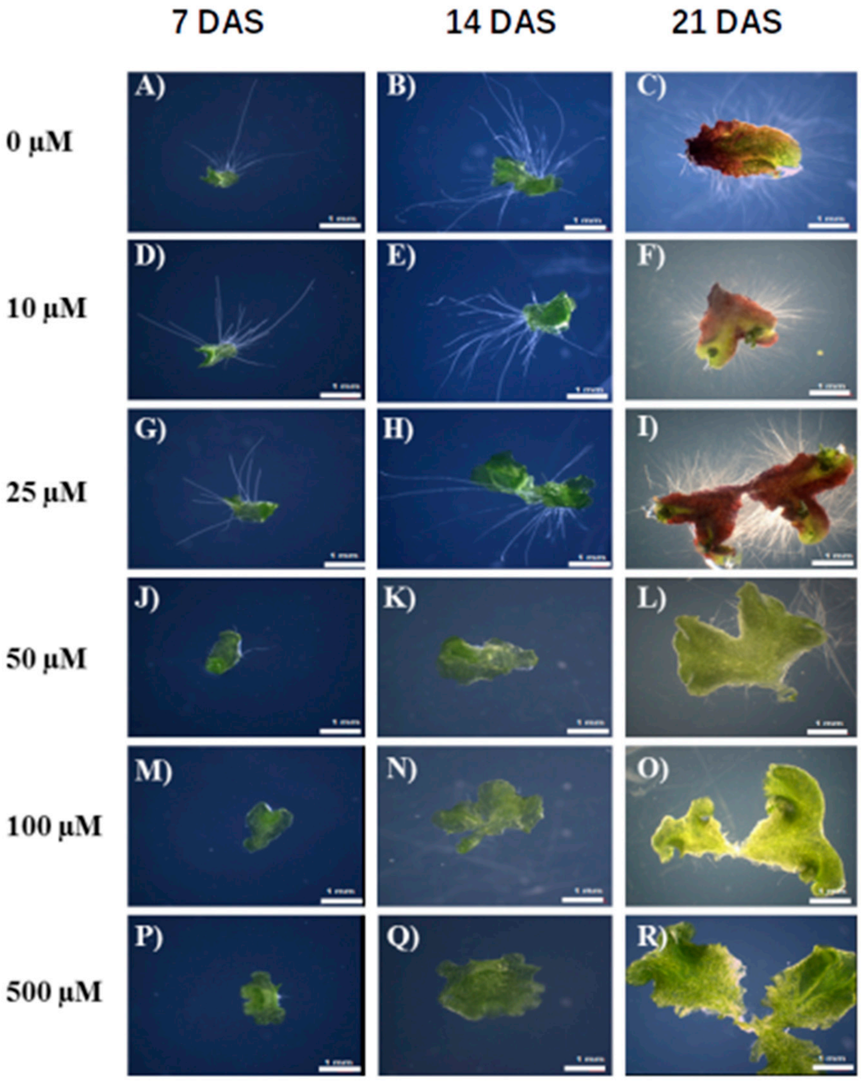

Supplementary figure 25. Phenotypical response to Pi availability on marchantia polymorpha. We sown gemmae on media MS supplemented with Pi at 0, 10, 25, 50, 100 and 500 µM. The effect of Pi availability on thallus development were registered by photographs at 7, 14 and 21 DAS.

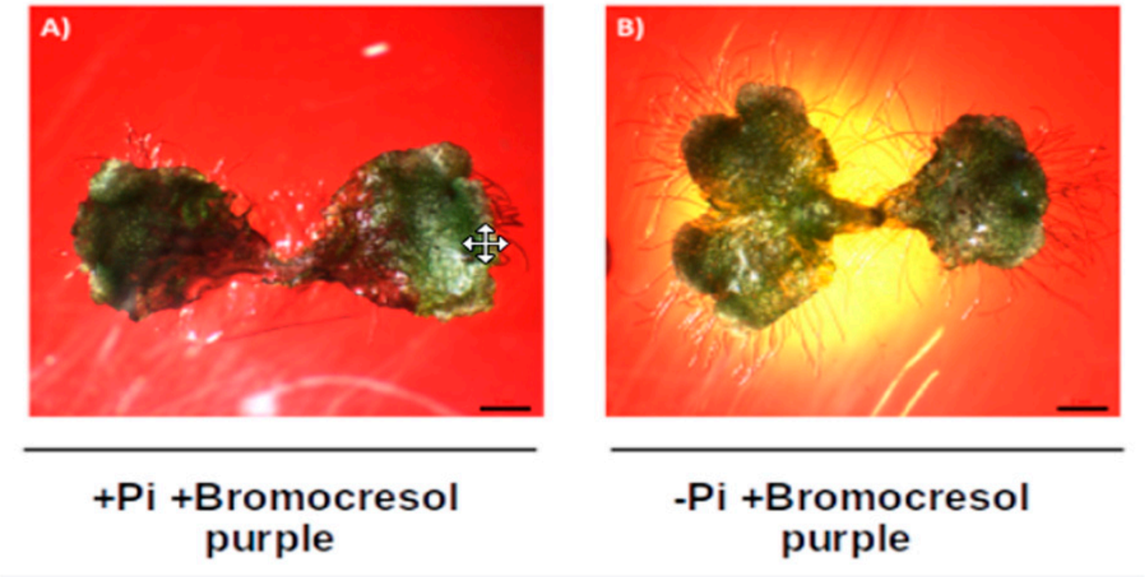

Supplementary figure 26. Thallus promotes media acidification under low Pi availability. We sown gemmae on media MS supplemented with +Pi (500 µM) and -Pi (10 µM) for 7 days. Then we transfer the thallus to the same medium supplemented with bromocresol purple at 5.7 pH (color red), we incubate overnight and then pictures were taken. A) Showed no change in color indicator for the thallus under +Pi. B) Showed a change from red to yellow which means the acidification in the area behind thallus and rhizoids in response to low Pi.

MpID(V5.1)	MpID(V3.1)	Gene	12 HPT	24 HPT	150 HPT
Mp1g25400	Mapoly0002s0332	MpMLS1	0	0	0
Mp6g10540	Mapoly0016s0095	MpMLS2	-1.59	1.39	-1.3
Mp2g11400	Mapoly0023s0108	MpMLS3	-26.86	-1.8	-4.88
Mp4g07200	Mapoly0115s0061	MpMLS4	0	0	0
Mp8g16290	Mapoly0154s0035	MpMLS5	-16.64	-1.35	-3.33
Mp8g09100	Mapoly0063s0010	MpCIS1	1.04	-1.91	1.35
Mp5g15980	Mapoly0071s0012	MpCIS2	-1.04	-1.89	4.31
Mp8g12790	Mapoly0083s0041	MpCIS3	0	0	0
Mp6g21480	Mapoly0091s0007	MpCIS4	0	0	0
Mp7g15800	Mapoly0111s0039	MpCIS5	-1.02	-1.39	1.7
Mp8g04310	Mapoly0200s0007	MpCIS6	-4.33	-35.18	-25.6
Mp8g04380	Mapoly0216s0011	MpCIS7	0	0	0
-	Mapoly0383s0001	MpCIS8	0	0	0
Mp8g04980	Mapoly0383s0002	MpCIS9	0	0	0
Mp3g01810	Mapoly0007s0172	MpALMT1	0	0	0
Mp7g05600	Mapoly0057s0111	MpALMT2	0	0	0
Mp4g14650	Mapoly0070s0016	MpALMT3	-1.54	-1.09	-1.13
Mp3g25480	Mapoly0100s0061	MpALMT4	-10.13	-9.58	-1.43
Mp1g28440	Mapoly0002s0036	MpMATE1	1.63	1.02	2.45
Mp7g12320	Mapoly0003s0243	MpMATE2	0	0	0
Mp1g04370	Mapoly0005s0170	MpMATE3	1.08	1.05	2.66
Mp1g11450	Mapoly0014s0081	MpMATE4	-1.16	-1.83	2.06
Mp6g11640	Mapoly0016s0203	MpMATE5	0	0	0
Mp3g05110	Mapoly0022s0017	MpMATE6	1.07	1.22	-2.56
Mp3g04300	Mapoly0022s0101	MpMATE7	0	0	0
Mp1g16390	Mapoly0033s0021	MpMATE8	-4.33	-3.43	3.03
Mp4g18440	Mapoly0041s0125	MpMATE9	-1.51	-1.3	-1.84
Mp4g18470	Mapoly0041s0128	MpMATE10	1.96	1.06	9.19
Mp6g15320	Mapoly0056s0043	MpMATE11	-1.22	-1.7	1.4
Mp2g22500	Mapoly0072s0081	MpMATE12	0	0	0
Mp5g00270	Mapoly0078s0029	MpMATE13	0	0	0
Mp6g21080	Mapoly0091s0047	MpMATE14	-1.07	-1.05	1.68
Mp6g02120	Mapoly0248s0004	MpMATE15	1.67	-2.42	6.81

Supplementary figure 27. Transcriptional regulation of genes involved in organic acid biosynthesis and exudation. The heatmap shows the transcriptional profile in log2FC of genes involved in malate/citrate synthase and transport. Orange color represents up regulated genes, black no change genes and blue means down regulated genes under Pi starvation.

MpID(5.1)	MpID(V3.1)	Gene	12 HPT	24 HPT	150 HPT
Mp2g23410	Mapoly0191s0011	MpPLD1	1.52	1.04	6.13
Mp7g11840	Mapoly0003s0195	MpPLD2	-1.07	-1.64	1.06
Mp7g11840	Mapoly0003s0195	MpPLD3	-1.07	-1.64	1.06
Mp1g07530	Mapoly0043s0145	MpPLD4	0	0	2.66
Mp5g16720	Mapoly0117s0034	MpPLD5	1.95	1.36	-1.53
Mp2g18270	Mapoly0177s0006	MpPLD6	0	0	0
Mp1g19150	Mapoly0001s0253	MpPLD7	-2.08	-6.42	-1.26
Mp2g17310	Mapoly0353s0001	MpPLD8	0	0	0
Mp2g17320	Mapoly4085s0001	MpPLD9	2.49	-1.44	-1.52
Mp2g10950	Mapoly0023s0061	MpMGDG	0	0	0
Mp5g12930	Mapoly0092s0015	MpDGDG	0	0	0
Mp3g04330	Mapoly0022s0098	MpDAG	-1.28	-1.08	1.66
Mp5g24070	Mapoly0010s0049	MpSQD	1.62	1.35	-2.43
Mp3g23340	Mapoly0024s0110	MpXPL	1.16	-1	-2.25
Mp3g21770	Mapoly0089s0039	MpPAH	-1.21	1.44	1.27

Supplementary figure 28. Transcriptional regulation of genes involved in lipid turnover. The heatmap shows the transcriptional profile in log2FC of genes involved in phospholipid degradation and sulpholipid/galactolipid synthesis. Orange color represents up regulated genes, black no change genes and blue means down regulated genes under Pi starvation.

MpID(V5.1)	MpID(V3.1)	12 HPT	24 HPT	150 HPT
Mp8g16260	Mapoly0154s0038	-7.84	-1.1	-1.1
Mp5g01660	Mapoly1623s0001	-1.26	-4.97	-4.72
Mp3g16820	Mapoly0039s0113	-1.19	-2.97	-1.42
Mp5g17110	Mapoly0196s0013	-2.56	-3.27	-58.06
Mp5g10730	Mapoly0048s0001	-12.6	-15.51	-10.03
Mp5g17030	Mapoly0117s0003	-5.05	-3.63	-15.49
Mp5g17250	Mapoly0182s0024	-3.82	-3.42	-65.73
Mp5g17480	Mapoly0182s0001	-1.22	-3.05	-4.29
Mp5g13830	Mapoly0032s0073	-4.12	-21.66	-1.13
Mp5g17260	Mapoly0182s0023	-4.27	-4.7	-16.67
Mp7g14900	Mapoly0009s0175	-1.05	-2.29	-7.29
Mp6g18300	Mapoly0038s0040	-1.54	-4.92	-4.62
Mp5g14530	Mapoly0032s0145	-3.45	-12.16	-1.57
Mp5g13820	Mapoly0032s0072	-2.84	-3.04	-30.14
Mp5g17150	Mapoly0196s0010	-1.59	-5.7	-3.59
Mp7g14950	Mapoly2709s0001	-1.23	-2.21	-5.91
Mp5g06870	Mapoly0136s0035	-4.23	-9.85	-17.29
Mp4g14270	Mapoly0070s0055	-2.3	-3.15	-6.02
Mp3g18420	Mapoly0486s0001	-1.99	-12.75	-7.76
Mp5g07230	Mapoly0332s0001	-4.71	-70.54	-5.73
Mp7g09310	Mapoly0068s0084	-1.48	-2.94	-2
Mp5g17590	Mapoly0084s0011	-1.28	-6.77	-4.92
Mp5g02850	Mapoly0124s0038	-1.68	-11.45	-3.25
Mp3g18410	Mapoly0341s0001	-1.61	-9.17	-9.85
Mp2g20950	Mapoly0040s0117	1.59	-4.81	-1.28
Mp8g01720	Mapoly0064s0027	1.15	-1.38	-1.87
Mp5g13790	Mapoly0032s0069	1.81	-2.03	-6.18
Mp6g13510	Mapoly0047s0002	2.8	-1.48	-1.52
Mp5g13650	Mapoly0032s0056	1.2	-5.1	-3.22

Mp5g01710	Mapoly0161s0033	1.82	-5.85	-9.74
Mp5g03300	Mapoly0133s0056	2.23	-1.95	-1.87
Mp5g13710	Mapoly0032s0061	1.43	-2.1	-2.71
Mp4g13870	Mapoly0070s0094	1.95	-3.42	-1.2
Mp5g01640	Mapoly0175s0024	1.4	-1.3	-10.4
Mp5g12120	Mapoly0143s0041	1.05	-5.14	-1.21
Mp5g04320	Mapoly0243s0001	1.42	-2.77	-4.71
Mp5g03200	Mapoly0124s0003	3.86	-7.82	-1.88
Mp5g04290	Mapoly0992s0001	3.24	-11.48	-15.68
Mp5g01630	Mapoly0175s0023	2.35	-2.28	-3.07
Mp3g22000	Mapoly0089s0017	1.12	-3.58	-6.7
Mp5g17140	Mapoly0196s0011	1.33	-4.9	-2.57
Mp6g13520	Mapoly0047s0003	2.08	-1.69	-7.1
Mp5g12110	Mapoly0143s0040	1.44	-1.43	-1.76
Mp5g02520	Mapoly0147s0045	1.6	-4.37	-6.88
Mp5g13840	Mapoly0032s0074	1.24	-3.26	-2.92
Mpzg00410	Mapoly0562s0001	1.57	-2.3	-2.07
Mp6g13550	Mapoly0047s0007	3.38	-2.97	-1.19
Mp5g14500	Mapoly0032s0143	1.06	-3.24	-1.55
Mp2g19200	Mapoly0128s0034	1.21	-34.97	-4.07
Mp6g13320	Mapoly0059s0017	-1.12	1.13	-2.65
Mp8g09900	Mapoly0008s0232	-1.86	-4.62	2.68
Mp7g07630	Mapoly0076s0031	-1.46	-1.22	1.62
Mp8g07500	Mapoly0013s0043	-1.13	-22.01	2.76
Mp5g02840	Mapoly0124s0039	-1.26	-9.94	1.36
Mp5g10690	Mapoly0048s0003	-1.07	-4.28	2.42
Mp2g19250	Mapoly0055s0126	-1.11	-14.83	3.63
Mp7g13980	Mapoly0009s0083	-1.07	1.06	2.48
Mp3g15330	Mapoly0004s0139	-4.56	1.04	4.42
Mp1g06830	Mapoly0043s0075	2.1	2.68	-2.93
Mp3g05740	Mapoly0006s0045	3.25	1.48	-1.43
Mp1g12440	Mapoly0019s0014	1.74	1.27	-1.77
Mp3g12240	Mapoly0050s0029	2.06	1.75	-5.36
Mp3g12230	Mapoly0050s0028	1.59	1.27	-2.77
Mp2g08960	Mapoly0015s0180	1.62	1.17	-1.33

Mp5g04000	Mapoly0141s0008	2.32	1.49	-2.12
Mp7g02680	Mapoly0088s0020	1.18	-1.05	2.2
Mp5g14510	Mapoly0032s0144	1.93	-1.04	3.68
Mp8g01700	Mapoly0064s0029	1.26	-2.83	11.86
Mp2g19210	Mapoly0128s0035	1.48	-19.37	2.35
Mp5g14490	Mapoly0032s0142	1.58	-3.8	3.85
Mp2g19260	Mapoly0128s0033	1.38	-39.63	1.07
Mp5g17600	Mapoly0084s0012	5.82	-6.42	2.18
Mp5g07120	Mapoly0136s0009	4.65	-1.51	21.22
Mp7g19380	Mapoly0067s0040	3.06	-1.74	3.87
Mp4g05810	Mapoly0087s0010	1.48	-1.88	8.98
Mp3g20340	Mapoly0258s0001	1.02	-1.07	1.9
Mp5g10700	Mapoly0048s0002	1.56	-2.69	22.67
Mp4g14580	Mapoly0070s0023	1.85	-1.87	1.51
Mp8g17560	Mapoly0030s0090	1.35	1.05	1.82
Mp3g20400	Mapoly0149s0005	2.12	1.47	1.04
Mp3g15810	Mapoly0004s0091	4.55	2.28	1.49
Mp4g16740	Mapoly0054s0141	2.48	2.15	3.21
Mp3g15800	Mapoly0004s0092	12.96	2.81	3.26
Mp7g15000	Mapoly0009s0184	2.5	1.33	6.04
Mp8g09590	Mapoly0008s0265	7.25	2.84	2.02
Mp7g15010	Mapoly0009s0185	20.56	4.7	102.52
Mp3g15790	Mapoly0004s0093	7.57	3.71	1.92
Mp7g07600	Mapoly0076s0034	1.11	1.14	2.92
Mp7g19390	Mapoly0067s0039	2.4	1.05	52.93
Mp1g09290	Mapoly0096s0070	1.81	1.77	2.15
Mp7g05770	Mapoly0057s0094	1.18	1.55	1
Mp5g04010	Mapoly0141s0009	5.71	2.76	2.08
Mp3g15780	Mapoly0004s0094	6.39	2.56	2.21
Mp4g20060	Mapoly0116s0008	3.81	0	0
Mp2g08710	Mapoly0015s0156	0	0	50.62
Mp3g25050	Mapoly0100s0018	0	0	47.44
Mp5g02610	Mapoly0124s0062	0	-13.69	4.93
Mp7g17080	Mapoly0051s0045	0	-4.35	0
Mp5g10750	Mapoly0417s0001	-2.15	-2.91	0
Mp5g12140	Mapoly0143s0043	-1.5	-5.15	0

Supplementary figure 29. Transcriptional regulation of peroxidases (MpPRXs) genes. The heatmap shows the transcriptional profile in log2FC of *MpPRXs* genes involved in ROS signaling. Orange color represents up regulated genes, black no change genes and blue means down regulated genes under Pi starvation.

MpID(V5.1)	MpID(V3.1)	Gene	12 HPT	24 HPT	150 HPT
Mp3g20340	Mapoly0258s0001	MpNOX1	1.02	-1.07	1.9
Mp7g00270	Mapoly0046s0097	MpNOX2	-11.36	-5.63	-2.77

Supplementary figure 30. Transcriptional regulation of NADPH oxidase (MpNOXs) genes. The heatmap shows the transcriptional profile in log2FC of *MpNOXs* genes involved in ROS signaling. Orange color represents up regulated genes, black no change genes and blue means down regulated genes under Pi starvation. Mapoly0046s0097

MpID(V5.1)	MpID(V3.1)	Description	12 HPT	24 HPT	150 HPT
Mp5g19050	Mapoly0073s0038	MYB14	-1.03	-2.04	7.01
Mp2g05850	Mapoly0021s0041	PPO (aurone synthase candidate)	1.14	1.17	1.56
Mp1g05200	Mapoly0005s0088	Phenylalanine ammonia-lyase-like	-1.31	-1.54	1.51
Mp5g01410	Mapoly0175s0004	Chalcone isomerase-Like	-1.83	-2.06	1.25
Mp2g07060	Mapoly0021s0159	Chalcone synthase	-1.52	-2.46	1.79
Mp5g10260	Mapoly0048s0047	Shikimate Ohydroxycinnamoyltransferase	1.17	-2.71	2.22
Mp1g05220	Mapoly0005s0086	Phenylalanine ammonia-lyase	2.15	-1.38	1.79
Mp8g08700	Mapoly0063s0049	Cinnamoyl-CoA reductase	-1.03	-4	2.57
Mp1g10150	Mapoly0014s0211	Phenylalanine ammonia-lyase	-1.34	-2.23	-1.7
Mp5g01150	Mapoly0197s0009	Probable flavonoid related CYP450	1.93	1.19	1.65
Mp2g05850	Mapoly0021s0041	Polyphenol oxidase	32.92	-45	6.3

Supplementary figure 31. Transcriptional regulation of genes related to aronidin biosynthesis. The heatmap shows the transcriptional profile under low Pi conditions of several genes involved in the biosynthesis of the auronidins. Orange color represents up regulated genes, black no change genes and blue means down regulated genes under Pi starvation.

MpID(V5.1)	MpID(V3.1)	Gene	Fwd	Rev
Mp8g10550	Mapoly0008s0168	MpLPR1	CGACTCGTGCTCGCCATTCTGA	GTGAGCTAGATGAGCACAGCAG
Mp1g27550	Mapoly0002s0123	MpSPX	AGCGACTGTTGAAGGAGCAT	GACCACTCTTTCCTCGCTTG
Mp7g11330	Mapoly0003s0147	MpPHR1	CATCCTGGTCGGAGTACGTT	CTCGGAGGCTGTAAAAGTGC
Mp8g16290	Mapoly0154s0035	MpMLS3	CTAATCGCCCTCCTTTTTCC	GACCACTCTTTCCTCGCTTG
Mp5g21210	Mapoly0058s0103	MpCIS1	AGCGACTGTTGAAGGAGCAT	GACCACTCTTTCCTCGCTTG
Mp6g07050	Mapoly1011s0001	MpCLE1	TATACAGCGCGAACAGATGC	ACCGTCGTAAGACTCGTGCT
Mp5g18050	Mapoly0084s0052	MpCLE2	ACGCATCTCGTGGACTTAGG	AATCCCCCGTGTTTAAGGTC
Mp6g11010	Mapoly0016s0139	MpACT1	CGAGCTTCCCGACGGTCAAG	ACATGGTCGTTCCTCCAGAC

Supplementary figure 32. Table for qPCR primers. Shows the MpID for both genome version 3.1 and 5.1, gene name and primer sequence used for qPCR validation.
